# Rational and evolutionary engineering of *Saccharomyces cerevisiae* for production of dicarboxylic acids from lignocellulosic biomass and exploring genetic mechanisms of the yeast tolerance to the biomass hydrolysate

**DOI:** 10.1186/s13068-022-02121-1

**Published:** 2022-02-27

**Authors:** Vratislav Stovicek, Laura Dato, Henrik Almqvist, Marie Schöpping, Ksenia Chekina, Lasse Ebdrup Pedersen, Anna Koza, Diogo Figueira, Freddy Tjosås, Bruno Sommer Ferreira, Jochen Forster, Gunnar Lidén, Irina Borodina

**Affiliations:** 1 The Novo Nordisk Foundation Center for Biosustainability, Technical University of Denmark, Kemitorvet, Building 220, 2800 Kgs. Lyngby, Denmark; 2 Department of Chemical Engineering, Lund University, P.O. Box 124, 221 00 Lund, Sweden; 3 Biotrend S.A., Biocant Park Núcleo 04, Lote 2, 3060-197 Cantanhede, Portugal; 4 Borregaard ApS, Hjalmar Wessels vei 6, 1721 Sarpsborg, Norway; 5 Present Address: River Stone Biotech ApS, Fruebjergvej 3, 2100 Copenhagen, Denmark; 6 Present Address: Chr. Hansen A/S, Boge Alle 10-12, 2970 Hørsholm, Denmark; 7 Present Address: Division of Industrial Biotechnology, Department of Biology and Biological Engineering, Chalmers University of Technology, 412 96 Gothenburg, Sweden

**Keywords:** Biorefineries, Industrial yeast, Xylose, Adaptive evolution, Dicarboxylic acids, Hardwood spent sulfite liquor, Next generation sequencing

## Abstract

**Background:**

Lignosulfonates are significant wood chemicals with a $700 million market, produced by sulfite pulping of wood. During the pulping process, spent sulfite liquor (SSL) is generated, which in addition to lignosulfonates contains hemicellulose-derived sugars—in case of hardwoods primarily the pentose sugar xylose. The pentoses are currently underutilized. If they could be converted into value-added chemicals, overall economic profitability of the process would increase. SSLs are typically very inhibitory to microorganisms, which presents a challenge for a biotechnological process. The aim of the present work was to develop a robust yeast strain able to convert xylose in SSL to carboxylic acids.

**Results:**

The industrial strain Ethanol Red of the yeast *Saccharomyces cerevisiae* was engineered for efficient utilization of xylose in a *Eucalyptus globulus* lignosulfonate stream at low pH using CRISPR/Cas genome editing and adaptive laboratory evolution. The engineered strain grew in synthetic medium with xylose as sole carbon source with maximum specific growth rate (µ_max_) of 0.28 1/h. Selected evolved strains utilized all carbon sources in the SSL at pH 3.5 and grew with µ_max_ between 0.05 and 0.1 1/h depending on a nitrogen source supplement. Putative genetic determinants of the increased tolerance to the SSL were revealed by whole genome sequencing of the evolved strains. In particular, four top-candidate genes (*SNG1*, *FIT3*, *FZF1* and *CBP3*) were identified along with other gene candidates with predicted important roles, based on the type and distribution of the mutations across different strains and especially the best performing ones. The developed strains were further engineered for production of dicarboxylic acids (succinic and malic acid) via overexpression of the reductive branch of the tricarboxylic acid cycle (TCA). The production strain produced 0.2 mol and 0.12 mol of malic acid and succinic acid, respectively, per mol of xylose present in the SSL.

**Conclusions:**

The combined metabolic engineering and adaptive evolution approach provided a robust SSL-tolerant industrial strain that converts fermentable carbon content of the SSL feedstock into malic and succinic acids at low pH.in production yields reaching 0.1 mol and 0.065 mol per mol of total consumed carbon sources.. Moreover, our work suggests potential genetic background of the tolerance to the SSL stream pointing out potential gene targets for improving the tolerance to inhibitory industrial feedstocks.

**Supplementary Information:**

The online version contains supplementary material available at 10.1186/s13068-022-02121-1.

## Background

Industrial biotechnology nowadays provides processes for production of many organic compounds from biomass as carbon source, but development of new processes broadening the spectrum of marketable products is needed to offer new sustainable solutions for provision of fuels, materials and energy [1–3]. As the production of chemicals from renewable resources constantly faces the challenges of competition with petrochemical industry, production cost is a critical issue. Industrial biorefineries are complex multi-step facilities for conversion of crude and variable biomass resources by breaking down and fractionating the biomass into its smaller constituent parts to increase the value of the original biomass feedstock [4, 5]. Microbial conversion of carbon substrates via fermentation of sugars released from the biomass into fuels and other valuable chemicals has become an integral part of modern biorefineries [6, 7]. The yeast *Saccharomyces cerevisiae* is one of the organisms of choice for biorefinery applications. Its robustness, acid tolerance, and ease of use makes *S. cerevisiae* an important industrial workhorse [8]. Large-scale processes for production of ethanol, a natural end product of fermentation in *S. cerevisiae* from first generation feedstocks such as corn starch or sugar cane have been established and used world-wide for decades [9]. For bulk chemicals, substrate costs typically represent the major part of the overall production cost. There is an increasing interest in using lignocellulosic biomass as the feedstock for industrial processes. This is due to its abundance but also the fact that there is no direct competition with food production using this feedstock [10]. *S. cerevisiae* faces two main challenges in processes using complex agricultural or forestry residues as a substrate for fermentation. First, this yeast is not naturally able to utilize pentose sugars that are abundantly present in lignocellulosic biomass as hemicellulose polymer. Second, in contrast to first generation feedstocks, lignocellulosic streams need to be pretreated to release sugars from the rigid polymeric substance, releasing also a spectrum of compounds negatively affecting growth and fermentation performance of the host organism [11]. Decades of intensive research have yielded strains that are able to metabolize xylose, either via xylose isomerase (XI) from various bacteria or via xylose reductase (XR)/xylitol dehydrogenase (XDH) pathways [12–15]. Although both strategies have their drawbacks, such as inferior kinetic properties of XI enzymes or the co-factor imbalance and xylitol accumulation of the XR-XDH pathway [16, 17], both have been successfully improved and implemented into industrial *S. cerevisiae* strains [18]. Yeast is also sensitive to inhibitors formed during the pretreatment of lignocellulose, such as organic acids (e.g., acetic acid released from the hemicellulose treatment), phenols, furans etc. [19]. To overcome the growth inhibitory nature of lignocellulosic hydrolysates, extensive metabolic and evolutionary engineering efforts are required to adapt yeast strains to second-generation feedstocks [20–24]. The resistance to the industrial feedstocks is a complex phenomenon, where multiple genes are involved [25]. Several studies have shown a contribution of overexpression of particular genes to specific detoxification mechanisms, such as resistance to weak organic acids [26], phenolic compounds [27, 28] and furan derivatives [29, 30]. Simultaneous effect of several genes in the resistance to multiple stresses has also been demonstrated [31–33]. Despite these advances the complex nature of the inhibitor cocktail and genetic determinants of the inhibitor resistance are far from fully understood. Identification of new target genes is desirable for future rational engineering strategies to develop novel robust strains.

Ease of genetic manipulation, currently even improved by the emergence and implementation of advanced genetic engineering tools [34], broadens the spectrum of bio-based products produced in yeast far beyond ethanol [35, 36]. *S. cerevisiae* has shown its potential to be a suitable host for production of many different chemicals, ranging from advanced biofuels and bulk or fine chemicals, with several of those being already commercialized [36, 37]. As the production of lignocellulosic ethanol has also been commercialized [18], a few proof-of-concept efforts of production of chemicals such as lactic acid [38], 3-hydroxypropionic acid [39] or advanced fuels such as isobutanol [40] and 1-hexadecanol [41] from xylose have been demonstrated and production of more is anticipated [42].

Dicarboxylic acids, such as succinic, malic and fumaric acids, have been recognized as important platform chemicals that can be obtained from biomass via biological or chemical conversion and serve as building blocks for a wide spectrum of other valuable compounds including bio-based polymers [43]. These dicarboxylic acids are central compounds in the carbon catabolism, and are also found as end products in yeast although at very low levels. However, the titers can be improved via metabolic engineering [44]. An important advantage when using engineered *S. cerevisiae* as the host for production of dicarboxylic acids is its tolerance to low pH. Under acidic conditions, the dicarboxylic acids occur in un-dissociated forms, which potentially reduces their recovery and downstream processing costs. Successful metabolic engineering strategies leading to relevant yields of dicarboxylic acids mainly from glucose in yeast have been applied [45, 46], with succinic acid being produced by an engineered yeast strain at industrial scale [47]. Recently, a study demonstrating production of l-malic acid from xylose in a laboratory fed-batch process has been reported [48].

In this study, the potential of yeast as cell factory for production of dicarboxylic acids from a waste biomass stream with xylose as the main carbon source was explored. A robust industrial *S. cerevisiae* strain was engineered for utilization of xylose and successfully evolved for tolerance to a hardwood spent sulfite liquor (SSL) at low pH. Production of malic and succinic acid was enabled by overexpressing the reductive route of the TCA cycle and was demonstrated from the xylose-rich biomass hydrolysate using the engineered tolerant strain. Potential genetic mechanisms of the strain tolerance to the SSL stream were investigated by means of next generation sequencing.

## Results

### Rationally engineered industrial strain consumes xylose in complex medium

**Fig. 1:**
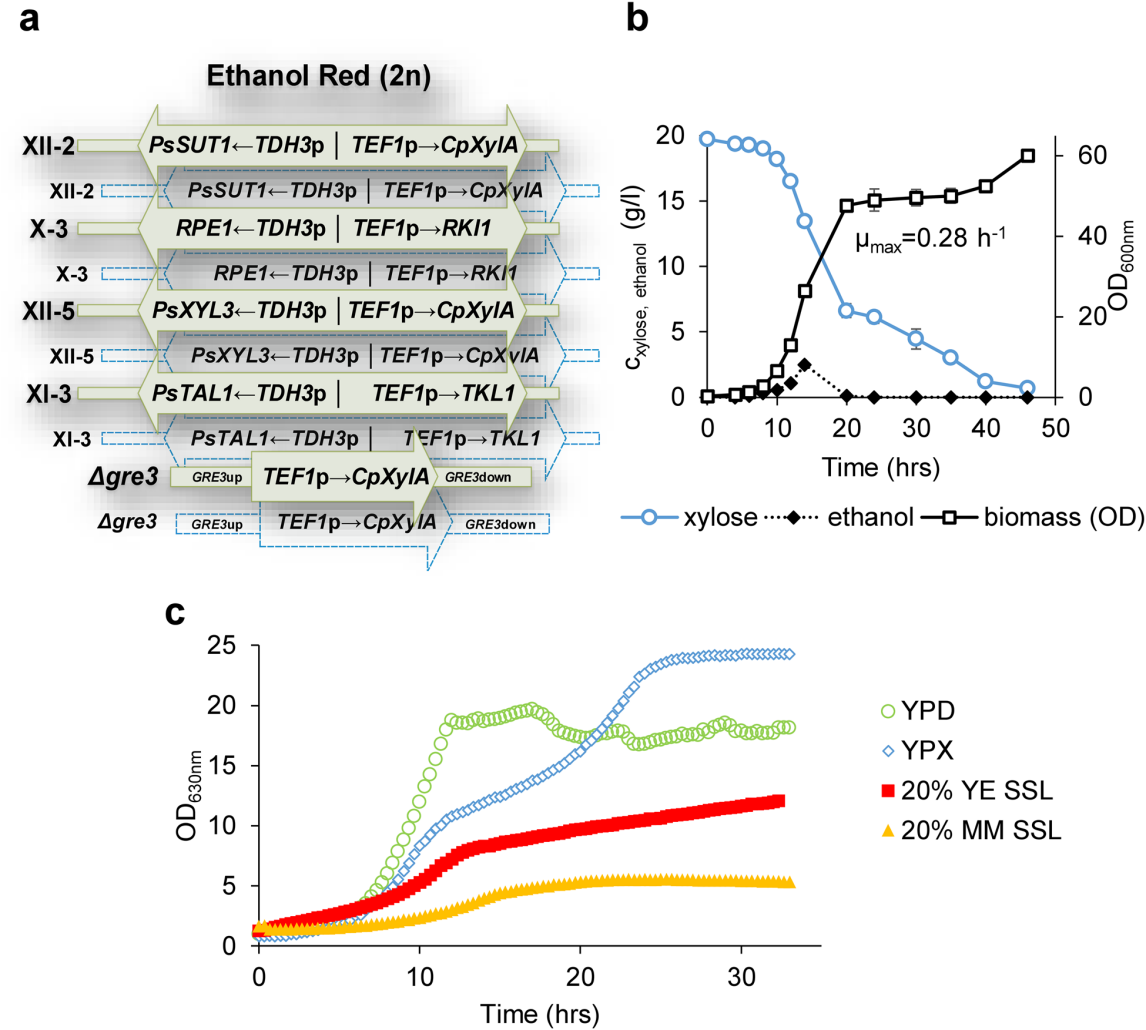
Engineering of an industrial *S. cerevisiae* strain for consumption of xylose. **a** Schematic illustration of gene expression modules integrated in the genome of the diploid (2n) industrial Ethanol Red strain. Expression of the integrated genes was driven by strong constitutive promoters as illustrated. The modules were integrated into the intergenic regions [111] (displayed on the left) or replacing *GRE3* ORF. *Ps* *Pichia stipitis*, *Cp* *Clostridium phytofermentans*, *XylA* gene for xylose isomerase. **b** Growth and xylose consumption of the XylC_2_ V1 strain in YPX in shake flasks. Error bars represent standard deviation (*N* = 2). **c** Comparison of growth performance of the rationally engineered strain in different conditions (standard complex media with glucose, YPD or xylose, YPX as carbon source, and the Eucalyptus SSL supplemented with yeast extract, YE or mineral medium, MM at pH = 3.5)

To develop a strain performing in the Eucalyptus spent sulfite liquor containing xylose as the major sugar (see “Methods” or [4]), the diploid industrial strain Ethanol Red, used in first generation bioethanol plants was engineered for xylose utilization. This was done via Cas9-mediated marker-less integration and overexpression of *P. stipitis SUT1*, encoding a sugar transporter with higher affinity for xylose, *P. stipitis XYL3* gene, encoding a D-xylulokinase, and four genes of the non-oxidative part of the pentose phosphate pathway (PPP): *RKI1*, *RPE1*, *TKL1* and a transaldolase encoding gene from *P. stipitis* (*PsTAL1*). The Cas9-mediated gene insertion ensured integration of each cassette into both targeted loci in the diploid genome (Fig. 1a). Most importantly, a codon-optimized xylose isomerase-encoding gene from *Clostridium phytofermentans* (*CpXylA*) [49] was thereby integrated into the genome in four copies to ensure higher level of somewhat poorly active enzyme in the cells (Fig. 1a). The CRISPR-Cas9 approach was also used for deletion of *GRE3* gene (encoding a reductase involved in formation of xylitol, which inhibits the activity of the xylose isomerase) with simultaneous integration of two additional copies of the xylose isomerase gene (Fig. 1a). A selected engineered strain (designated as XylC_2_ V1, Table 1) could grow in YPX medium with µ_max_ of 0.28 1/h and exhibited nearly complete consumption of xylose in less than 48 h (Fig. 1b). The xylose consuming strain was further tested for its growth performance in media containing the SSL feedstock. The Eucalyptus SSL was diluted into media containing either urea (mineral medium) or yeast extract as nitrogen source. Xylose was supplemented to the diluted SSL so that its total concentration was approximately 20 g/l. The pH of the media was adjusted to 3.5, to obtain a platform strain that would be suitable for low pH fermentation of the lignocellulosic feedstock, in line with the goal of developing a dicarboxylic acid producing strain. Low pH would allow for easier recovery of dicarboxylic acids directly from the fermentation broth and would avoid additional costs for downstream processing. The XylC_2_ strain could grow in both mineral medium-supplemented SSL (MM-SSL) and yeast extract-supplemented SSL (YE-SSL) with concentrations of the SSL around 20% v/v at the low pH, although the strain exhibited slower growth and reached lower OD values especially in the MM-SSL when compared to the growth profile in complex YPD or YPX media (Fig. 1c). Most likely, both the low pH and presence of inhibitors in the SSL had growth inhibition effects as pointed out previously [50]. Growth of the strain was significantly diminished in increasing concentrations of the SSL and virtually absent in concentrations exceeding 30% v/v and pH 3.5 in any tested conditions.

**Table 1:** List of strains used in the study

Strain ID (name)	Parental strain	Description
Ethanol red	–	*MATa/α*
XylC_2_ V1 (ST5415)	Ethanol red	*gre3Δ*::*TEF1p*-*CpXylA*/*gre3Δ*::*TEF1p*-*CpXylA* X-3: *TEF1p*-*RKI1* *TDH3p*-*RPE1* XI-3: *TEF1p*-*TKL1* *TDH3p*-*PsTAL1* XII-2: *TEF1p*-*CpXylA* *TDH3p*-*PsSUT1* XII-5: *TEF1p*-*CpXylA* *TDH3p*-*PsXYL3*
XylC_2_ EV1_1 (ST7042) XylC_2_ EV2_5 (ST7043) XylC_2_ EV4_5 (ST7044) XylC_2_ EV5_2 (ST7045) XylC_2_ EV6_4 (ST7046)	XylC_2_ V1	Single clone isolates from parallel lines evolved in the *Eucalyptus* SSL supplemented with yeast extract (YE-SSL)
XylC_2_ EV7_2 (ST7047) XylC_2_ EV8_2 (ST7048) XylC_2_ EV9_5 (ST7049) XylC_2_ EV10_1 (ST7050) XylC_2_ EV11_1 (ST7051) XylC_2_ EV12_2 (ST7052)	XylC_2_ V1	Single clone isolates from parallel lines evolved in the *Eucalyptus* SSL supplemented with mineral medium (MM-SSL)
XylC_2_ EV6_4 MA (ST7756)	XylC_2_ EV6_4	X-4: *TEF1p*-*SpMae1 TDH3p*-*sklΔMDH3* XI-1: *PGK1p*-*PYC2*

### Adaptive evolution yields tolerant phenotypes performing in the concentrated SSL

**Fig. 2:**
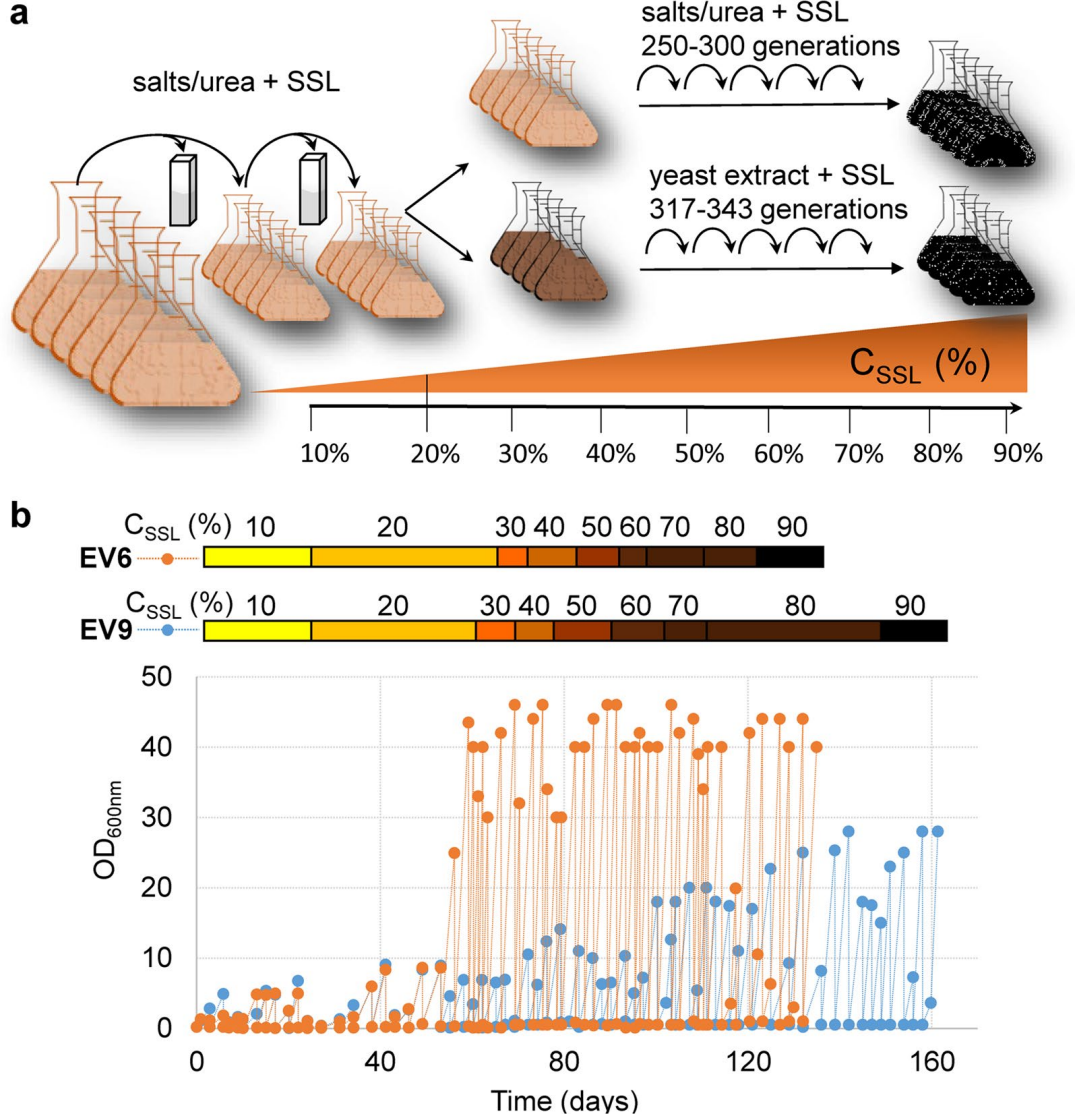
Adaptive evolution of the strain tolerance to the lignocellulosic biomass hydrolysate. **a** Schematic illustration of the evolution experiment. **b** Time-course of the evolution experiment, OD values of the culture during each transfer are displayed. A representative line from the YE SSL (EV6) evolution and a line from MM SSL (EV9) evolution are shown. Plots of all the remaining lines can be found in Additional file 2: Fig. S1. Increasing concentration of the SSL during the experiment is illustrated by the color bar

**Fig. 3:**
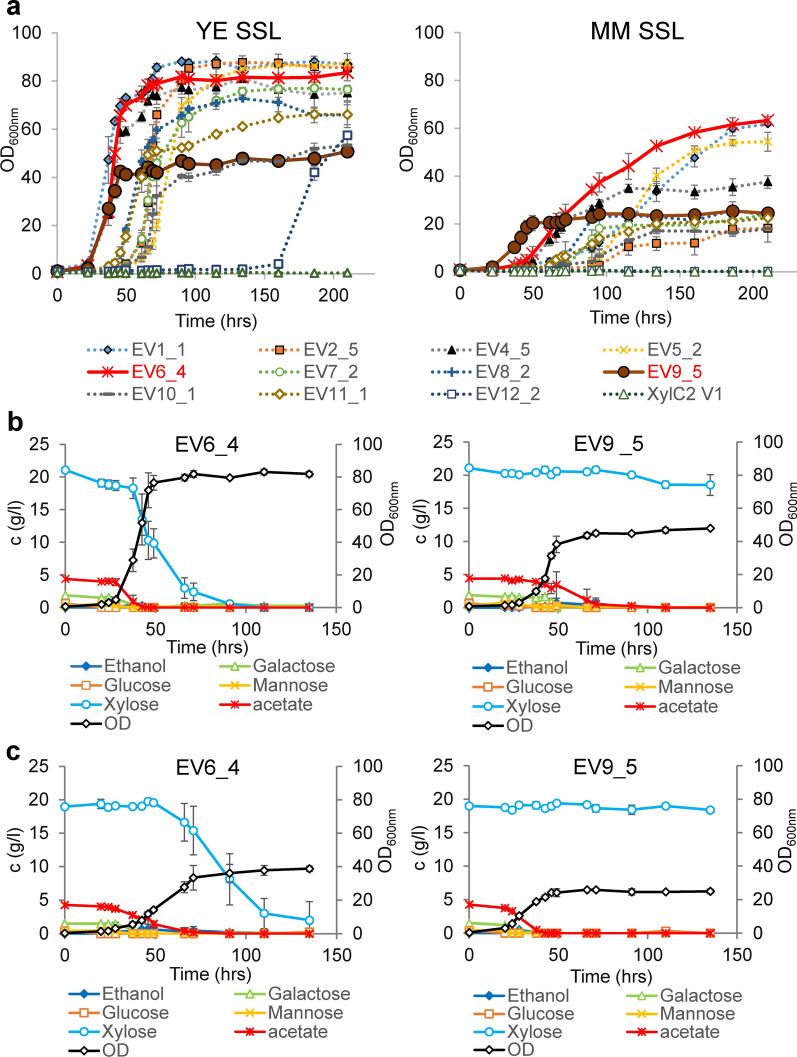
Growth and substrate consumption of the tolerant strains in the SSL. **a** Growth performance of the single isolates of each parallel evolution line in the concentrated SSL supplemented with either YE (left chart) or MM (right chart). The experiment was performed in two independent deep-well plates. Errors bars represent standard deviation (*N* = 2). **b** Growth and substrate consumption in the YE SSL by the selected evolved strains (XylC_2_ EV6_4 and EV9_5). The experiment was run in two parallel shake flasks. Error bars represent standard deviation (*N* = 2). OD values are plotted on the secondary *y*-axis. **c** Growth and substrate consumption in the MM SSL by the selected evolved strains. The experiment was run in two parallel shake flasks. Error bars represent standard deviation (*N* = 2)

To improve the strain tolerance to the feedstock, adaptive laboratory evolution was carried out in six parallel shake flasks via sequential batch transfers in increasing concentrations of the SSL (Fig. 2a). Initial conditions for adaptive evolution were set to 10% SSL (where significant growth was observed), supplemented with additional xylose, mineral medium and pH adjusted to 3.5. Selection of the medium with poorer (and cheaper) nitrogen source was made in the context with the planned process conditions. Nitrogen limitation would decrease the biomass yield and could provide more carbon for production of dicarboxylic acids. Particularly in the beginning of the evolution experiment, every new batch started with lower inoculum size (OD ≤ 0.1) to avoid the inhibitor tolerance given by large initial biomass concentration and allow for selection of adapted variants. Transfers to a fresh batch were usually made in the late-exponential phase, where no large increase in OD was seen. This corresponded to OD ~ 3 in early stages of the experiment in the diluted SSL, OD ~ 5 in > 30% SSL and OD ~ 10 of the cultures in > 50% SSL (Fig. 2b). The batch transfers in the diluted SSL did not lead to significantly increased biomass yields in any of the parallel cultures (Additional file 2: Fig. S1a). Therefore, the six parallel cultures were additionally diluted into the YE-SSL, after the cultures had been adapted for a few generations in the 20% MM-SSL, to support for variants with high biomass yield and presumably complete sugar consumption (Fig. 2a). As in case of the MM-SSL, the transfers were made in rather late exponential phase with the arbitrary bottom limit of OD ~ 20. When no further improvement of lag phase or biomass yield was seen, a passage to more concentrated SSL was executed in both parallel experiments (Fig. 2b). One of the parallel cultures (designated as EV3) from the MM-SSL experiment lost its growth activity after a transfer to the 70% SSL even when re-inoculated from a glycerol stock. Therefore, it was terminated and not included in further investigation (Additional file 2: Fig. S1b). After no significant improvement of biomass yields was seen in the concentrated SSL (~ 90%), the cells were streaked onto plates containing 60% SSL, and five randomly chosen colonies were selected to isolate single cell clones. After a preliminary screening showing similar performance of the single isolates within the particular evolution line, suggesting dominance of the phenotype in the population, one single isolate from each evolution line (designated as XylC_2_ EV1–EV12, Table 1) was characterized for growth in deep-well plates with the 80% SSL supplemented with either mineral medium or yeast extract at pH 3.5 (Fig. 3a). All the evolved isolates could grow in the YE-SSL. However, the growth was very different in several parameters comparing the different isolates. The YE-SSL-evolved variants grew faster (with µ_max_ between 0.08 and 0.1 1/h) and reached significantly higher OD than the majority of the MM-SSL-evolved variants. The one evolved strain with comparable length of the lag phase and µ_max_ 0.09 1/h was the strain EV9_5, yet reaching much lower final biomass concentration (Fig. 3a). On the other hand, several of the isolates performing very well in the YE-SSL grew poorly in the MM-SSL with very long lag phase (e.g., EV1_1, EV2_5). The variants with the shortest lag phase were EV4_5 and EV6_4, the latter reaching also high biomass concentration and µ_max_ 0.05 1/h. The MM-SSL-evolved variants grown in the MM-SSL also included strains with rather long lag phase reaching low biomass concentrations. However, the EV9_5 strain grew with the shortest lag-phase when compared to all the isolates and µ_max_ 0.05 1/h. The isolate EV12_2 showed a very long lag phase in the YE-SSL and did not grow in the MM-SSL under the given conditions, even though being evolved in the MM-SSL (Fig. 3a). Importantly, none of the strains lost the capability of growth in a synthetic medium with xylose as sole carbon source (Additional file 2: Fig. S2). The strains just slightly changed their growth profiles when compared to the parental XylC_2_ V1 strain, e.g., EV9_5 strain grew with the shortest lag phase but reached slightly lower OD values than the other strains. The majority of the YE-SSL-evolved isolates did not change the growth properties significantly, when, e.g., EV6_4 grew with a growth profile and biomass concentration comparable with the parental non-evolved strain (Additional file 2: Fig. S2).

Based on the growth evaluation in deep-well plates, strains EV9_5 (a representative of the MM-SSL evolution), exhibiting the shortest lag phase and highest specific growth rate among the MM-SSL-evolved variants, and EV6_4 (a representative of the YE-SSL evolution), a strain reaching high biomass concentration, comparable growth rate and slightly longer lag phase in the mineral medium-supplemented SSL, were selected for further characterization. The selected strains were grown in shake flasks in the concentrated (70%) SSL supplemented with either yeast extract or mineral medium at pH 3.5 and the carbon content consumption in the SSL was observed. While the EV9_5 strain consumed only hexoses and acetic acid, whereas almost the entire xylose content remained untouched, the EV6_4 strain consumed all the carbon sources including xylose in less than 90 h in the YE-SSL conditions, with xylose consumed last after all other carbon sources had been consumed (Fig. 3b). This explained the difference in biomass yields observed both in the deep-well plate growth comparison (Fig. 3a) and the shake flask experiment (Fig. 3b). At pH 4.5, similar growth parameters and carbon source consumption of the EV6_4 strain as at pH 3.5 were observed, with improved yet not complete consumption of xylose by the EV9_5 strain in the YE-SSL conditions (Additional file 2: Fig. S3a). A similar pattern was observed in the MM-SSL, just with lower growth rates and final biomass concentrations of both strains and virtually no xylose consumption of the EV9_5 strain at either pH 3.5 (Fig. 3c) or pH 4.5 (Additional file 2: Fig. S3b). Taken together, the results demonstrate that the evolution experiment yielded clearly different phenotypes in accordance with the complexity of the substrate, i.e., the conditions the strains were exposed to during the evolution. On one hand, a strain (EV6_4) was found to consume almost the entire carbon content although exhibiting slightly longer lag phase in the conditions with poorer nitrogen source. On the other hand, a strain (EV9_5) exhibited relatively short lag phase in the MM-SSL conditions but no (or poor) xylose consumption in the SSL, although the strain clearly retained the capacity to utilize xylose (Additional file 2: Fig. S2). However, it obviously lacked the capability of xylose consumption in the complex substrate perhaps due to partial oxygen limitation in later stages of growth in the shake flasks, when other carbon substrates had been consumed and biomass accumulated.

### Whole genome sequencing of the evolved strains reveals mutations behind the tolerance to inhibitors present in the SSL

**Fig. 4:**
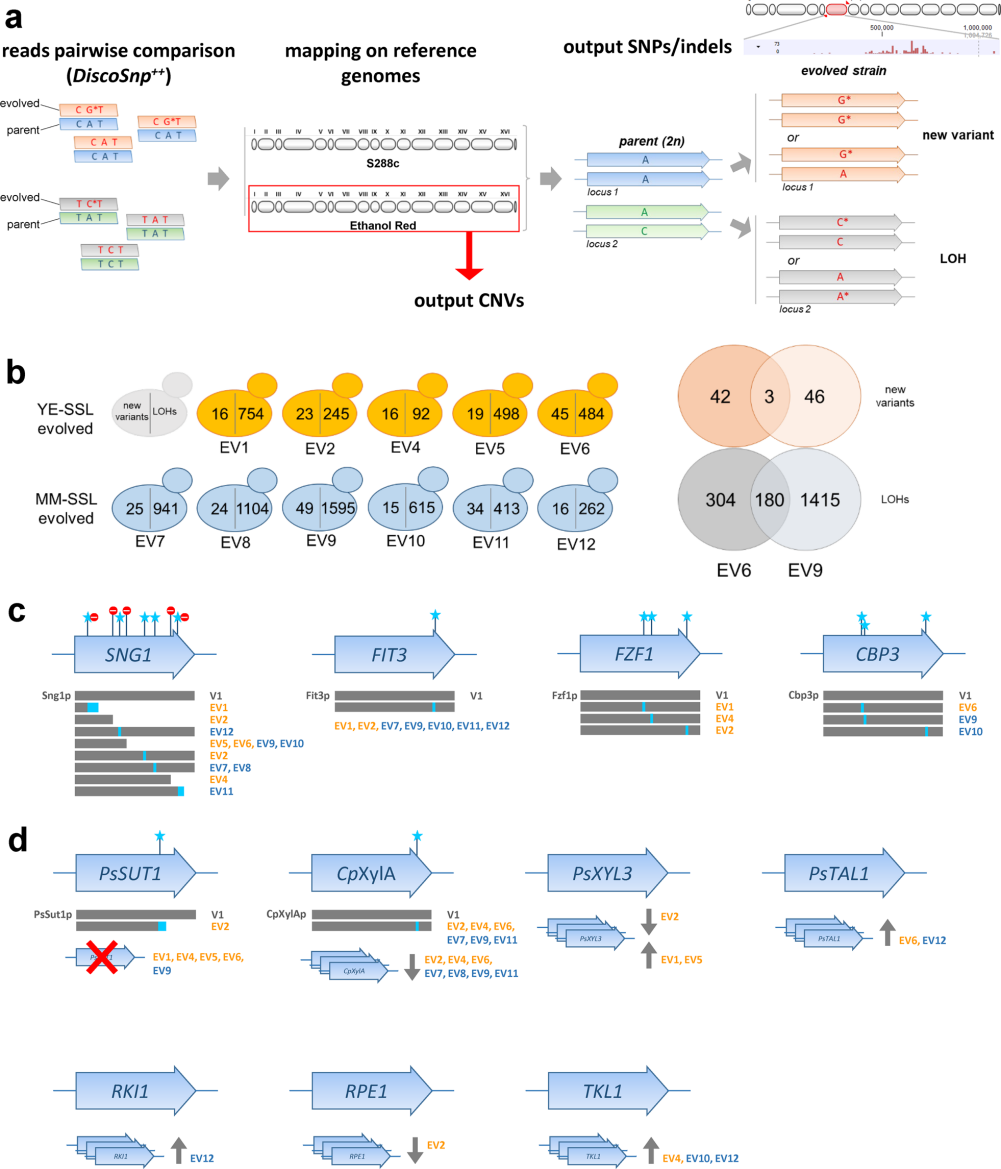
Next generation sequencing of the individual isolates evolved for tolerance to the SSL. **a** Schematic representation of the NGS data analysis and outcome. SNPs and indels were detected by reference-free pairwise comparison between each evolved isolate and the parental strain, performed with DiscoSnp +  + , followed by mapping of the variants to the reference genomes. CNVs were obtained by mapping to the Ethanol Red genome. **b** Number of mutations found per strain, split in new variants and loss of heterozygosity (LOH) events. Venn diagrams show overlaps between strains EV6_4 and EV9_5. **c** Schematic representation of the mutations found in some selected genes of interest, shared between strains. **d** Schematic representation of the changes found in the engineered xylose pathway genes

The genomes of all eleven single isolates from both evolution experiments, the parental rationally engineered strain and the wild type parent strain were sequenced with the Illumina NextSeq technology (“Methods” section). To identify small mutations, the software DiscoSnp^++^ was used to compare each strain with its direct progenitor, in a reference-free manner (Fig. 4a). This allowed on one side to account for the heterozygosity of the diploid strains and on the other side to overcome the difficulties related to the lack of a very high quality genome assembly for the parent strain, both factors otherwise resulting in an overall increase of false discovery rate during variant calling. The mutations were only subsequently mapped to the reference genomes (S288c and Ethanol Red). Data analysis of the DiscoSnp^++^ output was performed by a combination of bioinformatics tools, use of custom scripts and manual curation (see “Methods” section).

An overview of the results statistics is given in Table 2. Considering only the subset of mutations causing amino acid substitutions, 391 genes were affected in total (Additional file 1: Table S4). Of those, 172 genes were found mutated in both evolution experiments, i.e., in both the YE-SSL and MM-SSL media. Taking into account the diploid state of our strains, it was possible to distinguish between mutations that introduced a new variant and loss of heterozygosity (LOH) events. Of the total 2255 mutations found, we identified only 188 new variants (8.3%), while all the remaining were LOHs. Among the new variants, 103 caused amino acid changes distributed in 87 genes, 12 of which in common between the YE-SSL and MM-SSL media. About 55% of the new variants, therefore, caused an amino acid change, against the 28% of the LOHs. Interestingly, when mapping the variants on the different chromosomes, the vast majority of mutations resulted to fall within few chromosomal segments involved in LOH events (Additional file 2: Fig. S6), while only a relatively small number of isolated mutations was found. In particular, chromosomes VII, X and to a lesser extent XVI had large segments with high concentration of LOHs shared between strains of both YE-SSL and MM-SSL evolutions. Areas on chromosomes IV and VIII were involved in LOHs in strains from the MM-SSL evolution. Segments of chromosomes III and XIV underwent LOH only in strain EV2_5. It is highly probable that in each LOH segment, sometimes spanning half a chromosome or more, only one or few mutations were the “drivers” responsible for selection during evolution, while the rest was “hitchhikers” being inherited just as a consequence of the chromosomal rearrangement. For this reason, we decided to focus more on isolated mutations and new variants, and in general on mutations shared between the independent lines and/or occurring in the best performing strains. Figure 4b shows the number of mutations found per strain. EV6_4 and EV9_5 were the ones accumulating the highest number of new variants. EV9_5 also accumulated by far the highest number of LOHs, being, therefore, the most mutated strain with a total of 1644 mutations. EV4_5 was the strain with the lowest number of mutations, with a total of 108. It has to be noted, though, that LOH events include also reversions to wild type (or at least to the S288c-reference variants), which were for all strains roughly half of the LOHs.

**Table 2:** Number of small mutations (SNPs + INDELs) found in the evolved strains compared to the parent and mapped to either the whole reference S288c genome, the plasmid sequences that were introduced to engineer the parent strain to consume xylose, or the non-S288 genes which are present in Ethanol Red, respectively

	Total	In Reference *S. cerevisiae* Genome^a^			In inserted plasmids^b^			Ethanol Red-specific genes^c^		
		All	AA-changes	Genes with AA-changes	All	AA-changes	Genes with AA-changes	All	AA-changes	Genes with AA-changes
All mutations	2255	2188	663	380	26	2	2	40	20	8
In YE + SSL	1342	1290	405	246	23	2	2	28	14	5
In MM + SSL	1947	1899	554	307	26	1	1	22	9	4
Shared	1034	1001	297	170	23	1	1	10	3	1
New variants	188	177	101	85	8	2	2	2	0	0
In YE + SSL	98	90	57	48	7	2	2	0	0	0
In MM + SSL	116	106	56	49	8	1	1	2	0	0
Shared	26	19	12	11	7	1	1	0	0	0
LOH events	2067	2011	562	295	18	0	0	38	20	8
In YE + SSL	1244	1200	348	198	16	0	0	28	14	5
In MM + SSL	1831	1793	498	258	18	0	0	20	9	4
Shared	1008	982	285	159	16	0	0	10	3	1

*AA* amino acid

^a^S288c genome (R64, version GCF_000146045.2)

^b^Integrated xylose pathway

^c^ORFs that are not present in S288c

Enrichment analyses performed with the Term Finder tool of the *Saccharomyces Genome Database* revealed that among genes with amino acid-changing mutations shared between the two evolution experiments [i.e., common between YE-SSL and MM-SSL media (Additional file 3: Table S5)], GO Processes DNA integration (*p*-val 0.00021), RNA-dependent DNA biosynthetic process (*p*-val 0.00169) and RNA-mediated transposition (*p*-val 0.00740) were overrepresented. GO Functions enriched were DNA polymerase activity (*p*-val 6.21·10^–5^) and aspartic-type endopeptidase activity (*p*-val 9.32·10^–5^). All enriched terms were associated to transposable element genes. Considering genes with mutations causing amino acid substitutions in the best performing strain, EV6_4 (Additional file 3: Table S6), we found an enrichment in the GO Function signal transducer, downstream of receptor, with serine/threonine kinase activity (*p*-val 0.01731) linked to *STE11*, *FUS3* and *KSS1*. These genes encode three interconnected key components of the MAPK pathways regulating cell proliferation in relation to mating, pseudohyphal growth and cell wall integrity. *KSS1* carried an LOH mutation causing the non-conservative substitution P266T. Nearby mutations in Kss1p (D249G, E260G and D288G) were shown to confer a hyperfilamentous growth phenotype, through disruption of the binding with Ste12p [51]. *STE11* and *FUS3* carried new variant mutations causing amino acid substitutions A247T and A190E, respectively. In the same way we found for EV 9_5 (Additional file 3: Table S7) a significant enrichment in the GO Component membrane (*p*-val 0.01530, 43 genes) and the GO Function structural constituent of nuclear pore (*p*-val 0.04956), linked to genes *NUP192*, *NUP82*, *NUP57*, *NSP1*.

Looking for shared mutated genes with new variants, we found that all evolved strains had at least one mutation in *SNG1*, encoding an integral plasma membrane protein involved in multidrug resistance whose exact function is unknown (Fig. 4c; Additional file 2: Fig. S7a). The mutations were in most cases homozygous and led to truncation of the protein at various sites, except in three cases, where they caused non-conservative amino acid point mutations. It is likely that the activity of the encoded protein was disrupted or diminished as a result of most mutations. Another affected gene was *FIT3*, encoding a cell wall mannoprotein involved in the retention of siderophore–iron and, therefore, a facilitator of iron transport. Seven out of the eleven evolved strains were found to have the same heterozygous SNP (Fig. 4c; Additional file 2: Fig. S7b) causing the non-conservative mutation H32Q, located into a repeat region of the polypeptide. *FZF1*, encoding a transcription factor involved in sulfite metabolism, was found with different amino acid-changing SNPs in three out of the five strains evolved in YE-SSL: EV1_1, EV2_5 and EV4_5 (Fig. 4c; Additional file 2: Fig. S7c). The mutations fall within the 4th and 5th zinc-finger motifs of the protein. C162F is predicted to disrupt the structure of the 4th zinc finger, as it eliminates one of the two cysteine residues involved in zinc binding; H180N mutates the very same position that was previously shown to confer a sulfite-resistant phenotype to *S. cerevisiae*, by disruption of the 4th zinc finger [52]; D270Y is a non-conservative substitution and falls within the 5th zinc-finger. *CBP3,* encoding a mitochondrial chaperone required for assembly of cytochrome bc1, one the main components of the mitochondrial respiratory chain, was found with three different point mutations in strains EV6_4, EV 9_5 and EV10_1, including, therefore, the two best performing isolates.

Strain EV6_4 carried other interesting new variants which might have a role in its increased tolerance to SSL (described in Additional file 3: Table S8). Affected genes are *ERJ5*, *CRN1*, *FAP1*, *PCD1* and *TRM2*, the latter also found in three copies in all strains except EV2_5 and EV5_2. Another interesting new variant was found in EV9_5: *YPT1*, encoding a Rab GTPase involved in the secretory pathway, carried a mutation causing the substitution C23Y, where C23 is annotated as part of the GTP binding domain and also as a palmitoylated residue.

Some LOHs worth mentioning are the following. Genes *HAA1*, encoding a transcription factor with a prominent role in weak acid resistance, and *PDR12*, encoding an ATP-binding cassette transporter for weak organic acids, carried mutations causing the substitutions L186F and V1468K, respectively, in strains EV6_4, EV7_2, EV8_2. *ART5*, encoding a regulator of endocytosis and turnover of plasma membrane proteins, recently identified as implicated in spent sulfite liquor tolerance [53], carried mutations resulting in H13Y and K356M in strains EV1_1, EV6_4, EV9_5, EV10_1. *RCK1*, encoding a protein kinase involved in oxidative stress response whose overexpression was shown to improve acetic acid tolerance [54] carried a mutation causing truncation at position 88 of the protein in EV9_5.

Mutations were also found in the integrated sequences bearing the xylose metabolic pathway (Fig. 4d; Additional file 2: Fig. S8). *PsSUT1* was found with a homozygous deletion of four bases in strain EV2_5, causing a frame shift resulting in a stretch of 30 different amino acids followed by truncation of the last 138 amino acids of the protein (Additional file 2: Fig. S8a). A heterozygous point mutation was found in *CpXylA* in strains EV2_5, EV4_5, EV6_4, EV7_2, EV9_5 and EV11_1, causing mutation A388D in the protein. This position is not involved in the catalytic activity and the residue is located on the external surface of the protein, with its side chain facing outwards. The mutated amino acid is not predicted to interfere with folding of the protein (Additional file 2: Fig. S8b).

Analysis of copy number variation (CNV) revealed changes occurred in several of the evolved strains. First of all, there was no direct correlation between the changes in copy number and the LOH regions, but only some partial scattered overlaps. The only exception was on chromosome VIII, where the LOH region in EV12_2 corresponds to the loss of one copy of the left arm. None of the other strains showed partial chromosome losses. A large number of genes from chromosome IV were found in one extra copy in one or more strains of the YE-SSL evolution (mainly EV1_1, EV4_5, EV6_4). Genes were not from a circumscribed area but scattered along the chromosome, about 300 in total with limited overlap between strains: 73 were common between EV1_1, EV4_5 and EV6_4, which were also the best performing strains of the group, and only one, *SED1*, common between four out of the total five YE-SSL strains. *SED1* encodes the major stress-induced structural GPI-anchored cell wall glycoprotein, involved in cell wall organization and mitochondrial genome maintenance. When considering the subgroup of 73 common genes (Additional file 3: Table S9), we found significant enrichment in GO Processes organelle organization (*p*-val 6.91·10^–7^), chromosome organization (*p*-val 0.02003), regulation of signal transduction (*p*-val 0.04647) and in GO Function protein binding (*p*-val 0.00670). Chromosome III was found duplicated or partially duplicated in strains of the MM-SSL evolution, where EV9_5 carried two extra copies of most of its genes (and one extra of the remaining), EV7_2 and EV8_2 had one extra copy of all genes (two extra in very few cases), while EV10_1, EV11_1 and EV12_2 had one extra copy of part of them. Considering the genes duplicated in all MM-SSL strains (Additional file 3: Table S10), we found enrichment in GO Function oxidoreductase activity, acting on NAD(P)H, nitrogenous group as acceptor (*p*-val 0.00158), linked to two genes: *FRM2* and *HBN1*. The first one encodes an oxidoreductase that has a role in lipid signaling and is involved in oxidative stress response, and the second encodes a putative oxidoreductase which responds to iron depletion, DNA replication stress and is also linked to oxidative stress. Genes from chromosomes XI and a smaller number from chromosome I were found with one extra copy in many of the strains from both media evolutions (particularly EV9_5 with more genes involved). When considering a core set of genes which were common to most of the strains (excluding three strains with considerably less genes involved: EV2_5, EV5_2, EV11_1), (Additional file 3: Table S11), we found enrichment in the GO Process telomere tethering at nuclear periphery, (*p*-val 0.00948), GO Function structural constituent of nuclear pore (*p*-val 0.00608) and GO Component nuclear pore (*p*-val 0.00425). These terms were linked to genes *NUP133*, *NUP120, MLP1* and *LOS1*, all coding for components of the nuclear pore and involved in several processes including double-strand break repair, transcription, chromatin silencing and telomere length control. Some genes from chromosomes XV, XVI and V were also found with one extra copy in strains from both media evolutions, but not in EV9_5.

Regarding the engineered xylose pathway (Fig. 4d; Additional file 1: Table S12), the heterologous gene *PsSUT1* was deleted in strains EV1_1, EV4_5, EV5_2, EV6_4 and EV9_5. *PsXYL3* was present in only one copy instead of two in strain EV2_5, while strains EV1_1 and EV5_2 gained one more copy of it. Strain EV2_5 also lost two copies of *CpXylA*, thus remaining with four, and strains EV4_5, EV6_4, EV7_2, EV8_2, EV9_5 and EV11_1 lost one copy of it, thus remaining with five instead of six. Strains EV6_4 and EV12_2 gained one copy of *PsTAL1*. One extra copy of *TKL1* was present in EV4_5, EV10_1 and EV12_2. Strain EV12_2 gained also one copy of *RKI1*, and EV2_5 lost one copy of *RPE1*.

As shown above, most of the isolates rather retained the original phenotype regarding xylose utilization in defined conditions. This documents that none of the strains lost the ability to consume xylose as a result of the occurred genetic changes, as it was observed in other batch transfer adaptive evolutions [20].

### Engineered tolerant strain produces mixture of dicarboxylic acids in synthetic xylose media

**Fig. 5:**
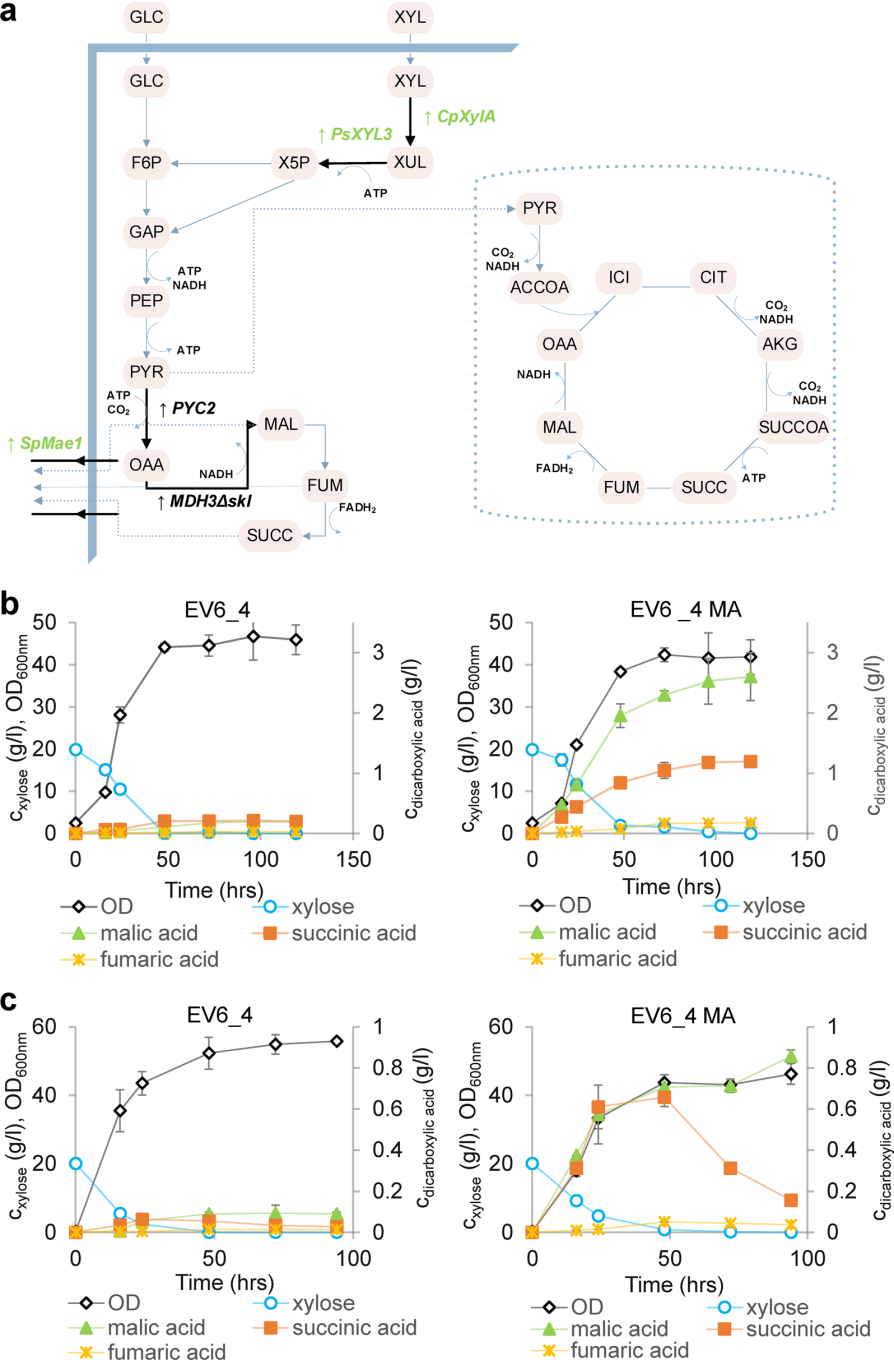
Production of dicarboxylic acids from xylose. **a** Schematic illustration of the metabolic pathways, where dicarboxylic acids are involved. Genes overexpressed in the evolved engineered strain are displayed. Engineered parts of the production pathway are highlighted by thick black arrows and heterologous genes displayed in green letters. **b** Dicarboxylic (malic, succinic, fumaric) acid production by XylC_2_ EV6_4 and EV6_4 MA strains in YEX medium with CaCO_3_ or **c** in YEX with no external buffer. Concentration of dicarboxylic acids is plotted on the secondary *y*-axis. The experiments were performed in duplicates, error bars represent standard deviation (*N* = 2)

The selected SSL-tolerant strain XylC_2_ EV6_4 was further engineered for production of dicarboxylic acids. This was achieved via overexpression of the reductive branch of the TCA cycle, which has been shown to be a strategy leading to enhanced production of particularly malic acid and no significant growth impair of the strains [46]. Following the strategy highlighted in Fig. 5a, a gene for dicarboxylic acid transporter from *S. pombe SpMae1* [55], the gene for native pyruvate carboxylase *PYC2*, and a gene encoding a malate dehydrogenase lacking peroxisomal localization signal *MDH3Δskl*, were integrated under the control of strong constitutive promoters into the genome of the evolved XylC_2_ EV6_4 strain. The strain designated XylC_2_ EV6_4 MA (Table 1) was grown in complex medium with xylose as sole carbon source. In addition, the strains were grown in the presence of CaCO_3_ that provided a carbonate buffer preventing a drop in pH caused by formation of carboxylic acids and kept the pH values during the experiment between 7 and 8. From this preliminary characterization, it was observed that the strain was able produce mainly malic and succinic acid from xylose in yields of 0.15 mol/mol and 0.076 mol/mol xylose, respectively, with low proportion of fumaric acid in yield 0.01 mol/mol (Fig. 5b). The yields and titers were significantly higher in the CaCO_3_ conditions, where also slower xylose consumption was observed. In the absence of CaCO_3_ (and any other buffer), yields of 0.05 mol malic acid/mol xylose, 0.042 mol succinic acid/mol xylose and 0.003 mol of fumaric acid/mol xylose were obtained (Fig. 5c). Besides, it was observed that the malic acid yield was more affected by pH and/or carboxylation than the yield of succinic acid.

### Tolerant engineered strain produces dicarboxylic acids from the sugar content in the SSL at low pH

**Fig. 6:**
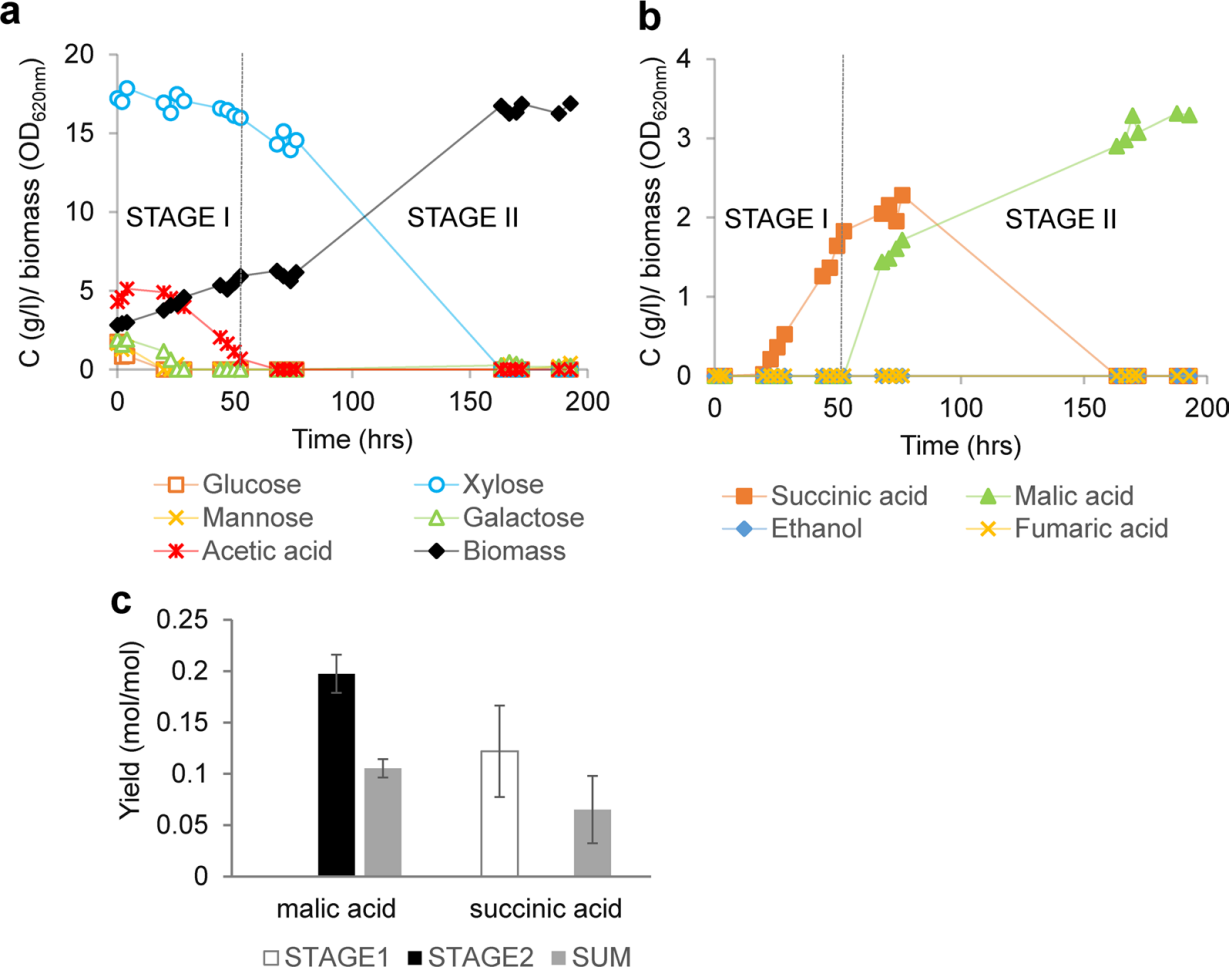
Production of dicarboxylic acids by fermentation of the SSL in bioreactors. **a** Consumption of the carbon sources present in the SSL and biomass formation by the engineered XylC_2_ 6_4 MA strain. **b** Production of dicarboxylic acids from the SSL at pH = 4.5. Due to the different length of the lag phase and subsequent time shift between the parallel bioreactors, a representative bioreactor experiment was chosen and is displayed. Two particular stages of the experiment characterized by consumption of hexoses, acetic acid and production of succinic acid (STAGE I), and xylose consumption and malic acid production (STAGE 2) are indicated. The data for the parallel bioreactor experiment is given in Additional file 2: Fig. S5. **c** Yields of dicarboxylic acids in mol of the formed product per mol of the carbon sources consumed. The yields are given for each stage as well as a sum of the both stages, i.e., endpoint titer of the product per total amount of consumed carbon. For succinic acid that is re-consumed during the second stage, the highest titer was taken for the yield calculation. The values are averages of both bioreactor experiments with standard deviation (*N* = 2)

The tolerant engineered strain EV6_4 MA was investigated for conversion of sugars contained in the SSL to dicarboxylic acids in bioreactors. As the growth of the EV6_4 MA strain, in terms of lag phase length, was clearly affected in more concentrated SSL when compared to the parental tolerant EV6_4 strain (Additional file 2: Fig. S4), 60% SSL was used as carbon source and supplemented with semi-defined medium containing nitrogen source. This corresponded to about 17 g/l of xylose as well as small amounts of the hexoses (galactose, glucose and mannose) (Fig. 6a), in total around 20 g/l of sugars and a *C*/*N* ratio ~ 10. In addition, the substrate contained about 4.5 g/l of acetate, originating from the SSL. As the production of dicarboxylic acids decreases with decreasing pH value [56], the pH of the experiment was controlled at a value of 4.5. To improve the dicarboxylic acid production, the sparging gas was enriched with CO_2_ to promote carboxylation of pyruvate to oxaloacetate by pyruvate carboxylase. The experiment was run in two parallel cultivations. The cultivations differed in the duration of the lag phases, but were otherwise similar (Fig. 6a; Additional file 2: Fig. S5). In the first stage of the experiment, only hexoses were consumed, resulting in cell growth as well as slight increase in acetate concentration (Fig. 6a; Additional file 2: Fig. S5). Later in this stage, acetate was consumed, whereas xylose was not consumed to any significant extent until acetate had been exhausted. Succinic acid was formed as the main product except for biomass during this stage (Fig. 6b; Additional file 2: Fig. S5). Xylose consumption took place in the second stage, where malate was formed (Fig. 6b) along with the significant increase of biomass formation (Fig. 6a; Additional file 2: Fig. S5). During this phase, re-consumption of succinate was also found (Fig. 6b; Additional file 2: Fig. S5). Yield of produced succinic acid was 0.12 ± 0.04 mol/mol of the carbon sources consumed in the first stage (Fig. 6c), and 0.065 ± 0.032 mol/mol of the total consumed carbon source. Malic acid yield was 0.2 ± 0.019 mol per mol of consumed xylose in the second stage, where malic acid production occurred, and 0.1 ± 0.009 mol/mol of the sum of all the carbon sources consumed (Fig. 6c). Fumaric acid or ethanol were not detected in any stage of the experiments.

## Discussion

A second-generation biorefinery application requires a strain efficiently converting waste and side streams rich in pentose sugars to added-value chemicals. Industrial *S. cerevisiae* strains are suitable hosts due to their robustness and low pH tolerance. Furthermore, they have proved their potential as biocatalysts performing in harsh industrial conditions in large-scale processes leading to production of biofuels from first as well as second-generation feedstocks [11, 18]. In this study, the industrial Ethanol Red strain, a bioethanol producer from first generation plants, was engineered for xylose utilization via the genomic integration of a xylose isomerase gene from *C. phytofermentans* [49], overexpression of genes encoding enzymes of the PPP and deletion of *GRE3*, diminishing byproduct xylitol formation [57]. Lignocellulosic biomass represents an abundant source of renewable organic matter. It involves a wide selection of raw materials from agricultural residues, energy crops to wood forestry biomass. Polymeric nature (cellulose, hemicellulose and lignin) and recalcitrance of the raw biomass requires harsh physicochemical deconstruction of the polymeric substance to release single constituents. However, existing pretreatment procedures also lead to formation of compounds severely inhibiting microbial growth and fermentation performance [4, 58]. In this study, an SSL from *Eucalyptus globulus* was used. Eucalyptus belongs among fast-growing hardwood trees that in contrast to softwood feedstocks contain high percentage of pentoses in the hemicellulose fraction. SSLs are a side product from acidic sulfite wood pulping used for separation of cellulosic fibers. The soluble lignosulfonates formed in the process have a wide range of uses or serve as source of lignin-based value-added products [59]. Besides lignosulfonates, SSLs contain also sugars released from hemicellulose that can be fermented as well as inhibitors, such as acetic acid, furfural, and phenolic compounds [60]. Use of xylose rich SSL streams for ethanol production by engineered *S. cerevisiae* [61] and natural xylose consumers such as *P. stipitis* [62] has been attempted. However, the presence of growth inhibitory compounds in these SSL streams represents a serious challenge for any microorganism. The engineered xylose consuming strain constructed here could not tolerate the SSL in concentration above 30% at pH 3.5. Low pH selection was made within the aims of the study, i.e., to obtain strains producing dicarboxylic acids from the SSL, as the downstream processing and product recovery usually significantly contribute to the total process costs and need to be considered when designing a viable process. In fact, the acids at pH values higher than their pKa are produced in their dissociated form and have to be converted into their acidic form by adding a strong acid. This results in by-product formation in terms of a salt, which is not desirable in large-scale production processes, and low pH processes are, therefore, considered to be more attractive [47]. Adaptive evolution, a genome-wide engineering approach, has become a commonly performed strategy for improvement of the tolerance to industrially relevant stresses [63], single inhibiting compounds present in lignocellulosic hydrolysates [64] or even complex mixtures of inhibitors and feedstocks as such [65]. Due to the complexity of the provided substrate and the putative synergistic effect of the inhibitor cocktail in the feedstock and presence of several stressors, the strain tolerance was in this study improved using sequential batch transfers in increasing concentrations of the SSL. The cells were challenged at a low inoculum size to avoid the inhibitor tolerance given by large initial biomass concentration [20] and allow for selection of adapted variants. Each transfer was made at late exponential/early stationary phase to target at survival variants and assumingly complete consumption of the carbon content in the feedstock. Interestingly, it has been found recently that early stationary populations had higher tolerance to a lignocellulosic feedstock at low pH [66]. Poorer nitrogen conditions when the SSL was supplemented with just salts and urea yielded only low biomass concentrations, due to no or incomplete consumption of the xylose content in the SSL. All the variants evolved in low nitrogen conditions failed in complete consumption of the carbon content even in nitrogen rich conditions. On the other hand, some of the strains evolved in the presence of rich nitrogen source reached higher biomass yields (and complete carbon consumption) even in conditions with poor nitrogen source, albeit with prolonged lag phase and in some cases even with rather poor growth rates. The SSL is a complex substrate containing several different carbon sources, which led to their sequential consumption. Hexoses, as usual for recombinant strains [67] were the preferred substrates as they were consumed in the first phase. In the second phase, acetate was consumed, which caused a rise in pH. During the third phase, xylose was consumed. The strategy promoting growth to high biomass yields turned out to be a successful approach for generating strains utilizing all the carbon sources even in poorer nitrogen conditions. The strains evolved in the poor nitrogen SSL conditions, although failing in complete xylose consumption in the SSL, clearly retained the capacity to utilize xylose. There was no loss of xylose utilization as reported in a previous study, where an evolution for inhibitor tolerance was performed [68]. Thus, these variants were evolved for survival in the presence of inhibitors and some of the best isolates even for shorter lag phase and presumably more efficient removal of inhibitory compounds in the SSL. Incomplete xylose consumption was observed perhaps due to partial oxygen limitation and lack of energy in later stages of growth in the shake flasks when hexoses had been consumed and biomass accumulated. Acetic acid presence clearly had an impact on the consumption of xylose as only when the broth was free of acetic acid, xylose consumption occurred. This could be explained by previous findings that acetic acid inhibits nutrient uptake [69], represents an energy burden and can thus also affect xylose uptake. Inhibitory effects of acetic acid on xylose fermentation in engineered *S. cerevisiae* strains have been demonstrated [70], being more severe with decreasing pH [71]. All the evolved strains significantly differed in the length of the lag phase. During the lag phase of fermentation, inhibitory compounds derived from the pretreatment step such as furfural, HMF and some phenolic derivatives can be converted by yeast cells (reviewed in [72]). Furthermore, the length of the lag phase was even more pronounced in more concentrated SSL and lower pH, affecting the total process time. This can be perceived as a trade-off situation spotted usually in evolutionary studies [65], when the strain with shortest lag phase was the one with no significant xylose consumption. However, the more important and successful aim of the adaptive evolution experiment was to obtain a strain capable of consuming all the carbon sources in process-relevant conditions, where the parental non-evolved strain could not grow at all.

Next generation sequencing revealed several genetic changes occurred in the evolved strains and mutations that might be good candidates putatively responsible for tolerance to the SSL. First of all, even though here we did not investigate structural changes occurred at the chromosome level, the evolved genomes presented a high rate of LOHs and CNVs that point to a high degree of chromosomal rearrangement. LOH distribution suggests that large chromosomal fragments were lost and replaced using the homologous chromosome as template. Only in one case, in strain EV12_2, the left arm of chromosome VIII was lost and remained in single copy. This partial aneuploidy might explain the phenotype of EV12_2, which performed poorly compared to the other evolved strains, particularly in MM-SSL: in fact, the affected region comprises genes whose hemizygous mutants are annotated as haploinsufficient, for example *DUR3*, encoding the urea plasma membrane transporter, and *QCR10*, coding for a subunit of respiratory complex III, among others. LOH can be considered as a stress response mechanism facilitating rapid adaptation as it allows for beneficial recessive variants to be expressed and generates novel allele combinations, having an important role in the evolution of yeast genomes [73–76]. Part of the rearrangements leading to LOHs might have been triggered by Ty elements, as suggested by the presence of Ty LTRs at some of the breaking points. In our evolved strains, the total number of single LOH SNPs/indels surpassed new variants (also termed de novo mutations) by at least an order of magnitude, making it very challenging to identify putative causative mutations among them, which we expect to be only a few per strain according to the number of events encompassing large chromosomal segments.

CNVs also play a major role in the evolution/adaptation of yeast to different environments [76, 77], and the large number of genes affected in the evolved strains of this study suggests the relevance of these changes under the harsh conditions imposed by the presence of multiple stressors with synergistic and pleiotropic effects. Unfortunately, it also further complicates the picture when trying to identify the most relevant determinants of tolerance. The scattered distribution of duplicated genes, even on chromosomes, where large areas were affected (including chromosome III which was affected as a whole), might suggest the involvement of circular intermediates in the amplification events, which can be triggered by transposable elements or other repetitive sequences or occur even in the absence of repeats [78–80].

We found an enrichment of transposon-related GO terms among the genes carrying amino acid-changing mutations in both YE-SSL and MM-SSL evolutions, similarly to what was reported previously after adaptive evolution of Ethanol Red to a combined lignocellulosic hydrolysate and heat stress [20], with seven genes overlapping between the two data sets. Our data do not provide information about transpositional activity. However, a recent study found an increased transcriptional activity for 77 transposable element genes in an industrial *S. cerevisiae* strain adapted to lignocellulosic environments [81], and among those are most of the transposon genes present in our data set. This finding, together with the chromosomal rearrangements found in our strains that might have been triggered by transposable elements, could be an indication that a similarly increased transpositional activity might have occurred also in our evolution experiment. It is worth noticing that a link to transposition activity was also suggested by the enrichment analysis regarding the best-performing strain, since activation of the *KSS1* MAPK cascade was shown to induce Ty1 transcription and retrotransposition [82]. Whether the mutations in transposon genes might have a role in their increased activity is currently unknown. Another interesting possibility could be that some of those mutations were a consequence, rather than a cause, of the high transpositional activity, as an increased mutation rate is associated both with double strand break repair [83, 84] and with high transcriptional activity [85].

Analyzing the mutations found, we identified four top-candidate genes which we think might have had a crucial role in the acquired tolerance to SSL-induced stress, based on their mutation pattern in our evolved strains and the functions of their encoded proteins: *SNG1*, *FIT3*, *FZF1* and *CBP3*. *SNG1* was implicated in resistance to nitrosoguanidine, 6-azauracil and other growth inhibitory compounds [86–88] conferred by its overexpression, probably due to permease activity as part of the pleiotropic drug resistance (PDR) network. Recently, it was also reported that *SNG1* is overexpressed in response to vanillin, one of the main phenolic compounds released by lignin breakdown, and its deletion caused growth defects under vanillin stress [89]. Taking these data into account, it seems counter-intuitive that an inactivation or altered functionality of Sng1p would confer an advantage under SSL stress. However, another study [90] demonstrated that *SNG1* expression increases plasma membrane fluidity through reduction of the flippase activity (which maintains the asymmetric distribution of phosphatidylserine and phosphatidylethanolamine between the inner and outer leaflets), via a complex mechanism involving several interactors and not yet fully elucidated. This is a very interesting finding and could represent a mechanism behind the acquired tolerance to SSL, since it is well known that several of the inhibitors, especially the weak acids, disrupt the membrane function by increasing its permeability. We speculate that disruption of Sng1p activity might contribute to counteract plasma membrane damage under SSL stress by modulation of its fluidity. *FIT3* was previously reported to be highly overexpressed in an industrial *S. cerevisiae* strain adapted to SSL when grown under SSL stress [91]. In another work, it was found upregulated in two *S. cerevisiae* strains under furfural stress, together with other siderophore–iron transport genes [92]. The authors’ hypothesis was a higher demand for iron under furfural stress, as iron–sulfur enzymes are known to be involved in response to oxidative stress. Since the structure of the protein is unknown as well as its mechanism of action, it is not possible to make specific hypotheses on the consequences of mutation H32Q. Regarding *CBP3,* due to its essential role for respiration and to the susceptibility of its null mutant to oxidative stress [93], mutations in its sequence might be highly relevant for SSL tolerance. For genes *SNG1, FIT3*, *FZF1* and *CBP3*, reverse engineering experiments should assess the role of the identified mutations in the parental background as well as in different strain backgrounds, which we imagine being positive towards the SSL tolerance. In addition, based on mutations found in the best performing strain, we suggest an effect in the establishment of SSL tolerance for genes *ERJ5*, *CRN1*, *FAP1*, *PCD1* and *TRM2*.

Overall, data from this work point to the putative involvement of a number of processes in the establishment of tolerance to the SSL at low pH: cell wall integrity (*STE11*/*KSS1* MAPK cascade and *SED1*); plasma membrane permeability (*SNG1*); iron availability (*FIT3*, *HBN1*); sulfite metabolism (*FZF1*); energy generation (*CPB3*); cytoskeleton organization and membrane trafficking (*CRN1*); protein folding (*ERJ5*); response to oxidative stress and detoxification of oxidized cellular components (*FAP1*, *PCD1*, *FRM2*, *HBN1*); DNA repair (*TRM2*, *HBN1*, *NUP133*, *NUP120*, *MLP1*) and transport through nuclear pore (*NUP192*, *NUP82*, *NUP57*, *NSP1*, *NUP133*, *NUP120*, *MLP1*).

Regarding the xylose pathway, we found some changes in the evolved strains which might have contributed to slight differences in the utilization of this sugar. First of all, five of the strains, including the best performing ones, lost the heterologous gene coding for the specific xylose transporter, and one strain had it truncated, indicating that the contribution of the transporter to xylose metabolism under the given conditions was negligible. Six of the evolved strains, including the best performing ones, were found to carry the same amino acid substitution in the xylose isomerase encoded by *CpXylA*. Although the mutation is not likely to have a direct impact on the folding or the catalytic activity of the protein, we cannot exclude an effect, for example on its physical interactions. Interestingly, the same strains carrying the mutation also had lost one copy (two in the case of EV2_5) of its coding gene. Some of the strains gained one copy of one of the other genes in the pathway (except for EV2 which lost one copy of two other genes). As stated above, none of the strains lost the ability to metabolize xylose anyway and their xylose utilization profile remained rather similar. Due to the role of the pentose phosphate pathway in regeneration of NADPH, important for detoxification of HMF and furfural [72, 94], differences in copy number of genes of this pathway could also have an impact on the lag phase.

The best SSL-tolerant strain was engineered for production of dicarboxylic acids via overexpression of the reductive pathway of the TCA cycle. The maximum theoretical yield of this pathway is two mol of malic acid per a mol of glucose. This engineering strategy led to the highest reported titers of malic acid from glucose in a laboratory strain in a previous study [46]. Here we showed production of malic and succinic acid from xylose in the engineered strain, with higher yields and titers at maintained high pH and presence of CaCO_3_ acting as the buffer and providing CO_2_ at the same time for the carboxylation step via the overexpressed pyruvate carboxylase, consistent with previous studies [46, 56]. An engineered laboratory strain used in the previous studies was a *pdc*^*−*^ strain lacking capability of pyruvate conversion to acetaldehyde to avoid loss of carbon towards ethanol. Such strain failed in growth on glucose and needed to be evolved for C2 source independency and glucose uptake [95]. Interestingly, apparently no or very little ethanol was produced in the engineered xylose strain growing in either synthetic xylose-rich media or in the SSL in the current work. This can be an advantage for production of malic acid from xylose when the xylose metabolism exhibits features of respiratory rather than fermentative metabolism [96] and no apparent need for additional metabolic and evolutionary engineering occurs. Weaker metabolic activity of lower glycolysis in case of growth on xylose is a likely interpretation of this phenomenon [96]. As mentioned above, high pH values are not suitable for the industrial production of dicarboxylic acids as the acids will be in their anion form and much alkali would be needed to neutralize the solution to efficiently recover the diacids from the fermentation broth. On the other hand, lower pH value also negatively impacts production of diacids, particularly malic acid due to thermodynamic export necessities and lowering of pH also significantly delays carbon source consumption [56]. Using lower nitrogen/carbon ratio and CO_2_ sparging we obtained production of both dicarboxylic acids with yield of malic acid around 0.2 mol per mol xylose in the SSL (and overall yield of 0.1 mol per mol of all the carbon sources). This is comparable to production yields of malic acid from glucose at low pH conditions [56]. Succinic acid production occurred earlier in the SSL with production yields of 0.065 mol per mol of the total carbon content and, as in non-buffered synthetic medium with xylose was re-consumed later on.

Some more focused additional metabolic engineering efforts to narrow down the product portfolio, e.g., downregulating competing pathways limiting production of byproducts or employing compound-specific transporters can still be performed [44, 46]. However, this is the first study reporting production of dicarboxylic acids from a xylose-rich biomass stream with yields of malic acid corresponding to the highest reported production of the product from glucose in defined laboratory conditions. Here, the sugar content of a xylose-rich waste biomass hydrolysate was converted into dicarboxylic acids by a robust industrial yeast cell factory.

## Conclusions

The use of industrial wastes for production of fuels and valuable chemicals is an important aspect of biomass valorization. Acidic sulfite wood pulping primarily separates lignin from cellulose and produces SSL streams that also contain fermentable hemicellulose-derived sugars. Using combined metabolic and evolutionary engineering effort, we developed industrial *S. cerevisiae* strains capable of dealing with the hardwood SSL and utilizing its sugar content at low pH. Moreover, we documented putative genetic determinants of the SSL tolerance and suggested potential targets for future improvement of tolerance to harsh industrial streams. We further demonstrated the conversion of the sugars into malic and succinic acid by the engineered tolerant strains in conditions that are relevant for industrial settings taking into account also the product recovery. This study provides an attractive prospect for use of the yeast *S. cerevisiae* in a second-generation biorefinery application for production of platform chemicals from xylose-rich biomass feedstocks.

## Methods

### Strains

All the yeast strains used and constructed in this study (Table 1) were derived from the diploid industrial *S. cerevisiae* strain Ethanol Red (obtained from Fermentis, a Lesaffre division, France). *Pichia stipitis* strain LY 1321 was used as the source for *P. stipitis* genes, *Schizosaccharomyces pombe* strain CBS 356 as the source of a *S. pombe* gene. *Escherichia coli* strain DH5α was used as a host for cloning and plasmid propagation. *E. coli* cells were grown at 37 °C in lysogeny broth (LB) containing 100 mg/l ampicillin or 50 mg/l kanamycin.

### Media

Yeast cells were grown in standard complex yeast extract/peptone (YP) medium with either 20 g/l glucose (YPD) or 20 g/l xylose (YPX) at 30 °C. The media were supplemented with 20 g/l agar for preparation of a solid medium. For selection, 200 mg/l G418 sulfate, 200 mg/l hygromycin B, or 100 mg/l nourseothricin were added.

Evolution experiments were performed in the SSL from *Eucalyptus globulus* (batch number DP-2018, provided by Borregaard ApS, Sarpsborg, Norway). The main components in the SSL were: 26.5 g/l xylose, 1.6 g/l mannose, 2.4 g/l glucose, 2.2 g/l galactose, 1.1 g/l arabinose, 9.1 g/l acetic acid. The SSL was supplemented with either mineral medium (MM) that was composed of 5 g/l of urea, 6 g/l KH_2_PO_4_, 6.6 g/l K_2_SO_4_, 0.5 g/l MgSO_4_.7 H_2_O, vitamins and trace elements as described in [97] or 10 g/l yeast extract (YE). For pH control, 50 mM potassium hydrogen phthalate in combination with HCl or NaOH was used. Xylose was supplemented in concentration of 20 g/l or 10 g/l to all SSL media with less than 30% or 50% of the SSL, respectively.

Production of dicarboxylic acids by engineered strains was performed in media containing yeast extract and 20 g/l xylose (YEX) with initial pH set to 7. For the calcium carbonate experiments, initial pH of the YEX medium was set 4.8 and added to shake flasks with CaCO_3_ in a final concentration of 10 g/l.

The bioreactor experiments were performed in media containing a mix of 1 g/l of yeast extract and 1.15 g/l of urea as nitrogen source giving the *C*/*N* ratio = 10, and trace minerals and vitamins (provided as 1.7 g/l of yeast nitrogen base without amino acids and ammonium sulfate, Amresco). 60% (v/v) Eucalyptus SSL was used as carbon source. For seed cultures 30% SSL was added to YPX medium and pH adjusted to 4.5.

### Adaptive evolution

The engineered xylose consuming strain XylC_2_ V1 was grown overnight in YPX medium and diluted into six parallel 250 ml-shake flasks containing 50 ml of mineral medium-supplemented SSL (MM-SSL) (10%) with pH adjusted to 3.5. Sequential batch transfers in the medium with the increasing concentration of the SSL were performed, diluting the cultures to a fresh batch of the medium. OD values at 600 nm of the cultures were determined using a spectrophotometer (Nanophotometer P300 Pearl, Implen) at each transfer. Additional six shake flasks with yeast extract-supplemented SSL (YE-SSL) at pH 3.5 were included after the strains had been adapted to the 20% MM-SSL. After the cultures had reached the SSL concentration of 90%, an aliquot from each evolution line was taken and streaked onto agar plates with the 60% YE-SSL and pH 4. Single cell colonies from each evolution line were isolated.

### Cultivation conditions

Basic characteristics of the engineered strains were observed in 250-ml shake flasks with the working volume of 50 ml and 250-rpm agitation. OD values of the culture were measured using a spectrophotometer (Nanophotometer P300 Pearl, Implen) in standard 1-ml cuvettes. The initial growth experiments in different media and the diluted SSL were run in microtiter plates (transparent 96 well, Greiner Bio-One) in 150 µl volume with the OD values determined in 20-min intervals using a microplate reader at 630 nm (ELx808, Biotek). Quantitative growth comparison of the engineered xylose consuming strain and its evolved progeny in YPX medium was performed using the Growth Profiler 1152 (Enzyscreen, the Netherlands). Briefly, overnight cultures were centrifuged, washed and used for inoculation of polystyrene 24-roundwell microplates to the initial OD 0.3 in a final volume of 750 µl. The Growth Profiler was set to 225-rpm agitation and plate scanning in 15-min intervals. Generated cell density images were used for calculation of G-values that were converted into OD_600nm_ values using a calibration curve. Growth experiments in the concentrated SSL were performed in deep-well plates in 2-ml volume with 300-rpm agitation. OD values of the cultures were determined using a spectrophotometer after dilution. The experiments with the dicarboxylic acid-producing strains were done in 250-ml shake flasks in 50-ml volume, in case of the calcium carbonate conditions the cells were mixed with the medium and added to a shake flask with CaCO_3_.

### Bioreactor conditions

For bioreactor experiments, 2.5 l bioreactors with a 1 l working volume (Braun Biostat C and Sartorius Biostat A +) were used. Bioreactors were operated at 30 °C, stirred at 600 rpm by dual rushton turbines and pH was controlled at 4.5 with 5 M KOH. The reactors were sparged with a mixture of air and CO_2_ (50 vol% of each), at a total flow rate of 0.4 vvm. Bioreactors were inoculated with cells harvested from a shake flask seed culture (250 ml liquid volume). The seed culture was centrifuged and the cell pellet was resuspended and transferred to the bioreactors to give an initial cell dry weight of 1.5 g/l.

### Plasmid construction

The integrative vectors (Additional file 1: Table S1) were constructed by USER fusion [98]. The particular BioBricks (Additional file 1: Table S2) were amplified by PCR with Phusion U polymerase (ThermoFisher Scientific) under the following conditions: 98 °C for 2 min, 30 cycles of 98 °C for 10 s, 54 °C for 10 s, 72 °C for 30 s/1 kb, 72 °C for 10 min. Used templates and primers are listed in Additional file 1: Tables S1 and S3. DNA fragments were gel purified and incubated in HF buffer together with USER enzyme (New England BioLabs) for 25 min at 37 °C, followed by incubation at 25 °C for 25 min. The reactions were transformed into chemically competent *E. coli* cells. Empty integrative vectors were digested with FastDigest *SfaAI* (ThermoFisher Scientific) restriction endonuclease, nicked with *Nb.BsmI* (New England BioLabs) and assembled with PCR amplified genes and promoter(s) of choice. Heterologous genes were either synthetized by GeneArt or amplified from the genomic DNA of the organisms of origin (Additional file 1: Table S2). Single gRNA vectors were constructed via the whole plasmid amplification as described in [99] using the primers listed in Additional file 1: Table S3. Multiple gRNA vectors were constructed via assembly of several single gRNA expression cassettes into a USER site containing 2-µm vector as described in [100].

### Strain construction

Before yeast transformation, the integrative vectors were linearized by FastDigest *NotI* (ThermoFisher Scientific) restriction enzyme. All strains were initially transformed with the *Cas9-*carrying centromeric vector (Additional file 1: Table S1) prior to further modifications. For gene integration into a genomic location, a gRNA helper vector (see the list in Additional file 1: Table S1) targeting Cas9 to the particular genomic site was transformed along with the corresponding linearized template as described in [101]. *GRE3* ORF was replaced by an expression cassette containing the *CpXylA* gene driven by the constitutive *TEF1* promoter flanked with overhangs homologous to the *GRE3* upstream and downstream regions. Yeast cells were transformed by the standard PEG/LiAc method according to [102]. The cells were plated on selective YPD (or YPX) plates with the appropriate selection after 2–3 h recovery phase. The plates were incubated typically for 3–5 days. Verification of correct integrations was done by colony PCR using OneTaq^®^ Hot Start Quick-Load^®^ 2X Master Mix (New England Biolabs) using the manufacturer’s protocol and primers listed in Additional file 1: Table S3.

### Next generation genome sequencing

Yeast genomic DNA was extracted using Quick-DNA™ Fungal/Bacterial Miniprep Kit (Zymo Research, USA) from overnight liquid cultures according to the manufacturer’s protocol. The genomic libraries were generated using the TruSeq^®^ Nano DNA HT Library Prep Kit (Illumina Inc., USA). Briefly, 100 ng of genomic DNA diluted in 52.5 µl TE buffer was fragmented in a Covaris E220 ultrasonicator (Covaris, USA) with 5% duty factor, 175 W peak incident power, 200 cycles/burst, and 50-s duration under frequency sweeping mode at 5.5 to 6 °C. The ends of fragmented DNA were repaired by T4 DNA polymerase, Klenow DNA polymerase, and T4 polynucleotide kinase (ThermoFisher Scientific, USA). The Exo-minus Klenow enzyme was then used to add an ‘A’ base to the 3’ end of the DNA fragments. The adapters were ligated to the ends of the DNA fragments, and the DNA fragments ranging from 300 to 400 bp were recovered by beads purification. Finally, the adapter-modified DNA fragments were enriched by 8 cycles PCR. Final concentration of each library was measured by the Qubit^®^ 2.0 Fluorimeter and Qubit DNA Broad range assay (ThermoFisher Scientific, USA). Average dsDNA library size was determined using the Agilent DNA 1000 kit on an Agilent 2100 Bioanalyzer (Agilent Technologies, USA). Libraries were normalized and pooled in 10 mM Tris–HCl (pH 8.0) and 0.05% Tween 20 to the final concentration of 10 nM. Pool of libraries was denaturated in 0.2 N NaOH and neutralized in 200 mM Tris–HCl (pH 7.0). 1.3 pM pool of libraries was spiked with a 1% PhiX control and loaded onto the flow cell provided in the NextSeq 500/550 Mid Output v2 Reagent kit (300 cycles) and sequenced on the NextSeq platform (Illumina Inc., USA) with paired-end reads protocol and read lengths of 2 × 150 nt.

### NGS data analysis

Illumina reads of genomes of the parental Ethanol Red strain, engineered xylose consuming strain and the evolved variants were de novo assembled and aligned to the *S. cerevisiae* reference genome using Bowtie2 [103]. Variant calling was done using DiscoSNP +  + [104] with the following settings: [k = 31/101, D = 100, P = 100, b = 0]. Python was used to filter out variant calls which were shared between the parental and evolved strains. Due to a large number of variant calls, focus was put on variants that were likely to affect phenotype, specifically variants in protein coding sequences (CDS). This was done using the DiscoSNP function to map variants to a reference genome (but not using that reference for finding variants). The variants were mapped against both the S288C reference genome R64 (assembly version GCF_000146045.2, GenBank ID: 285798) and the Ethanol Red assembly [105]. Python and biopython [106] were used to determine which variants were present in CDS regions and whether they would cause a change in the amino acid sequence (e.g., single amino acid change or frameshift). Integrative Genomics Viewer [107] and CLC Genomic Workbench (version 11.0.2, Qiagen Bioinformatics) were used to visualize variants mapped on the reference genomes, compare the different strains and manually evaluate mutations. To investigate the sequencing coverage distribution across the genome, the sequencing reads were mapped using BWA–MEM (version 0.7.15–r1140) [108] with the –a setting and the results were converted to bam and sorted and indexed using SAMtools (version 1.3) [109]. Then the sequencing depth was calculated with SAMtools depth using the –aa setting to get all positions. The bam files and the calculated depths of coverage were used for plotting the coverage for each sample. This was done using the Gviz package (version 1.14.7) [110] in R (version 3.2.1). For each scaffold and plasmid in the reference, the annotation, genomic position and coverage were plotted using the GeneRegionTrack, DataTrack and plotTracks functions. The coverage values were adjusted by dividing the count by the median count for the sample and multiplying by two, thus converting coverage into an approximate copy number. In addition, the coverage was converted to Wiggle Track Format (WIG) files. These were constructed by writing the previously mentioned adjusted coverage values to a bedGraph format file using R and then convert these to WIG format using a python script. WIG files were visualized and manually evaluated with CLC Genomic Workbench.

### HPLC analysis

Concentration of xylose and other metabolites in culture supernatants was determined by HPLC. Xylose and other sugars were analyzed using Aminex HPX87P column (300 × 7.8 mm, 9 µm) at 85 °C. The mobile phase was Milli-Q water with a flow of 0.6 ml/min. Detection was done by Refractive Index (RI) detector. For organic acids, Aminex HPX87H ion exclusion column (300 × 7.8 mm, 9 µm) at 60 °C with 10 mM H_2_SO_4_ as the mobile phase at a flow of 0.6 ml/min. Acetate and ethanol were detected by RI-detector. Succinic and malic acid were detected by UV detector at 210 nm and fumaric acid detected by UV detector at 254 nm. The data were acquired and analyzed with Chromeleon software (Thermo Scientific) or Empower Ver3 software (Waters). Alternatively, malic acid quantification was performed spectrophotometrically using a malic acid assay kit (Megazyme) using the manufacturer’s protocol.

## Supplementary Information

**Additional file 1: Table S1.** List of vectors used in the study. **Table S2.** List of DNA BioBricks used in the study. **Table S3.** List of primers used in the study. **Table S4.** Genes with aminoacid mutations found across all strains. **Table S12.** Average copy numbers for the engineered xylose pathway genes in each strain.

**Additional file 2: Fig. S1.** Adaptive evolution of the xylose consuming strain XylC_2_ V1 in the SSL, parallel lines. Time course of each parallel evolution line. Color bars indicate increased concentration of the SSL. **a** MM SSL evolution lines. **b** YE SSL evolution lines. Asterisk indicates the EV3 evolution line that was terminated for poor tolerance in the 70% SSL. **Fig. S2.** Evolution of the xylose consuming strain, single isolates. Growth profiles of the evolved strains cultivated in YPX medium. **Fig. S3.** Evolution of the xylose consuming strain. Growth of the selected SSL-tolerant strains XylC_2_ EV6_4 and EV9_5 in the **a** YE SSL and **b** MM SSL at pH = 4.5. The experiment was performed in duplicates, error bars represent standard deviation (*N* = 2). **Fig. S4.** Growth of the tolerant XylC_2_ EV6_4 and its dicarboxylic acid producing derivative XylC_2_ EV6_4 MA in 70% and 60% YE SSL. The experiments were performed in two parallel shake flasks, error bars represent standard deviation (*N* = 2). **Fig. S5.** Production of dicarboxylic acids by fermentation of the SSL in bioreactors, second parallel bioreactor experiment. Consumption of the carbon sources present in the SSL and biomass formation by the engineered XylC_2_ 6_4 MA strain (on the left) and production of dicarboxylic acids from the SSL at pH = 4.5 (on the right). **Fig. S6.** Mapping of the mutations to the respective loci on the reference genome of S288c. Panel A: overview of the distribution across the whole genome (Chr I to XVI, top) of the total mutations (blue, bottom) and the aminoacid-changing mutations (red, middle) found across all strains. Panels B–R: Distribution of the variants, mapped to each individual chromosome, in each of the strains (V1 parent and EV1–EV12, indicated on the left). Mutations are highlighted in orange if heterozygous or in red if homozygous, and referred to the S288c genomic sequence (annotation is shown on top, genes are represented by green boxes. Only few gene names are visible due to limited space). Dashed blue boxes highlight chromosomal segments involved in LOH events. **Fig. S7.** Highlights of mutated genes. Distribution and position of the variants in each strain (V1 parent and EV1–EV12, indicated on the left). Mutations are highlighted in orange if heterozygous or in red if homozygous. Corresponding outcomes of each mutation on the protein are indicated. **a** *SNG1 / YGR197C*. **b** *FIT3 / YOR383C*. **c** *FZF1 / YGL254W*. **Fig. S8.** Results of the mutations found in the engineered xylose pathway genes. **a** *PsSUT1*: Alignment of the wt and the mutated protein sequence. Residues are labeled by Rasmol colors. The red box highlights the mutated residues up to the truncation site. **b** *CpXylA*: snapshot of a detail of the 3D structure obtained by homology modeling (https://swissmodel.expasy.org/) of the mutant A388D compared to the wt (accession no. A9KN98). Residues are colored according to charge. Position 388 is highlighted by green spheres.

**Additional file 3: Tables S5–S11**.

## Data Availability

The next generation sequencing data files have been deposited in Sequence Read Archive (SRA) of the NCBI under the accession no. PRJNA718744 and can be accessed using the following link: https://www.ncbi.nlm.nih.gov/bioproject/PRJNA718744.

## Abbreviations

CRISPR-Cas: Clustered regularly interspaced short palindromic repeats-CRISPR associated
HMF: Hydroxymethylfurfural
NGS: Next generation sequencing
SSL: Spent sulfite liquor
TCA: Tricarboxylic acid cycle
XDH: Xylitol dehydrogenase
XI: Xylose isomerase
XR: Xylose reductase
