# Supplementary material for: Rational and evolutionary engineering of Saccharomyces cerevisiae for production of dicarboxylic acids from lignocellulosic biomass and exploring genetic mechanisms of the yeast tolerance to the biomass hydrolysate
Source: Biotechnol Biofuels Bioprod. 2022 Feb 27;15:22. doi: 10.1186/s13068-022-02121-1 (PMC8882276; doi:10.1186/s13068-022-02121-1)
Supplement: Supplementary file 1 — Additional file 1: Table S1. List of vectors used in the study. Table S2. List of DNA BioBricks used in the study. Table S3. List of primers used in the study. Table S4. Genes with aminoacid mutations found across all strains. Table S12. Average copy numbers for the engineered xylose pathway genes in each strain. [file 13068_2022_2121_MOESM1_ESM.docx]

**Additional file 1**

**Table S1. List of vectors used in this study.**

| **Plasmid name** | **Integration site/**  **replicon** | **Yeast selection marker*** | **Expression cassette** | **Parental vector** | **Cloned BioBricks** | **Source** |
| --- | --- | --- | --- | --- | --- | --- |
| *EasyClone marker-free integrative vectors* | | | | | | |
| pCfB3034 | X-3 | - | - | - | - | [1] |
| pCfB3035 | X-4 | - | - | - | - | [1] |
| pCfB3036 | XI-1 | - | - | - | - | [1] |
| pCfB2904 | XI-3 | - | - | - | - | [1] |
| pCfB3039 | XII-2 | - | - | - | - | [1] |
| pCfB2909 | XII-5 | - | - | - | - | [1] |
| *Cas9 and gRNA helper vectors* | | | | | | |
| pCfB2312 | CEN/ARS | kanMX | *P_TEF1_*-*Cas9* | - | - | [2] |
| pCfB2311 | 2µ | natMX | gRNA targeting *ADE2* gene | - | - | [2] |
| pTAJAK-71 | 2µ | natMX | - | - | - | [3] |
| pCfB3496 | 2µ | hphMX | gRNA targeting *ADE2* gene | pCfB3496 | - | This study |
| pCfB5197 | 2µ | hphMX | gRNA targeting *GRE3* gene | pCfB2311 | - | This study |
| pCfB3589 | 2µ | natMX | gRNA targeting X-4 integration site | - |  | [1] |
| pCfB3041 | 2µ | natMX | gRNA targeting X-3 integration site | - |  | [1] |
| pCfB3045 | 2µ | natMX | gRNA targeting XI-3 integration site | - |  | [1] |
| pCfB3048 | 2µ | natMX | gRNA targeting XII-2 integration site | - |  | [1] |
| pCfB3050 | 2µ | natMX | gRNA targeting XII-5 integration site | - |  | [1] |
| pCfB3043 | 2µ | natMX | gRNA targeting XI-1 integration site | - |  | [1] |
| pCfB3526 | 2µ | natMX | 3 gRNAs targeting X-3, XI-3 and XII-2 integration sites | pTAJAK-71 |  | This study |
| pCfB5190 | 2µ | natMX | 2 gRNAs targeting X-4 and XI-1 sites | pTAJAK-71 |  | This study |
| *Vectors for metabolic engineering* | | | | | | |
| pCfB5520 | X-3 | - | *RPE1*←*P_TDH3_*-*P_TEF1_*→*RKI1* | pCfB3034 | BB1232, BB1233, BB0464 | This study |
| pCfB5521 | XI-3 | - | PsTAL1←TDH3p- TEF1p→TKL1 | pCfB2904 | BB0263, BB1234, BB0464 | This study |
| pCfB5522 | XII-2 | - | PsSUT1←TDH3p-  TEF1p→CpXylA | pCfB3039 | BB1235, BB1237, BB0464 | This study |
| pCfB5523 | XII-5 | - | PsXYL3←TDH3p-TEF1p→CpXylA | pCfB2909 | BB1235, BB1236, BB0464 | This study |
| pCfB5183 | X-4 | - | MDH3^ΔSKL^←TDH3p-TEF1p→SpMAE1 | pCfB3035 | BB0537, BB0546,  BB0464 | This study |
| pCfB5184 | XI-1 | - | PGK1p→PYC2 | pCfB3036 | BB0150, BB0009 | This study |
| *Vectors carrying promoters* | | | | | | |
| p1977 (pUC19-PTDH3-PTEF1) | ori | - | ←TDH3p-TEF1p→ | - | - | [4] |
| pSP-GM1 | Ori, 2µ | KlURA3 | PGK1p→ | - | - | [5] |

* All the vectors carry amp^R^ cassette for selection in *E. coli*.

**Table S2. List of DNA BioBricks used in this study**. All the BioBricks were generated by PCR.

| **Name** | **Descriptive name** | **Primer pair for PCR** | **Template for PCR** |
| --- | --- | --- | --- |
| BB0599 | BBhphMX | ID399USERrev, ID400USERfwd | synthetic gene [4] |
| BB0464 | ←P_TDH3_-P_TEF1_→ | PTEF1->_U2_rv, PTDH3_rv | p1977 (pUC19-PTDH3-PTEF1) |
| BB0009 | P_PGK1_→ | PPGK1_fw, PPGK1_rv | pSP-GM1 |
| BB0263 | *TKL1*_2→ | tkl1_U2_fw, tkl1_U2_rv | *S. cerevisiae* genomic DNA |
| BB1232 | *RKI1*_2→ | RKI1_2FW, RKI1_2REV | *S. cerevisiae* genomic DNA |
| BB1233 | *RPE1*_1← | RPE1_1FW, RPE1_1REV | *S. cerevisiae* genomic DNA |
| BB1234 | *PsTAL1*_1← | TAL1_1FW, TAL1_1REV | *P. stipitis* genomic DNA |
| BB1235 | *CpXylA*_2→ | XIclos_2FW, XIclos_2REV | synthetic gene [4] |
| BB1236 | *PsXYL3*_1← | XYL3_1FW, XYL3_1REV | *P. stipitis* genomic DNA |
| BB1237 | *PsSUT1*_1← | PsSUT1_1FW, PsSUT1_1RE | *P. stipitis* genomic DNA |
| BB0546 | *SpMae1*→ | SpMae1_U2_fw, SpMae1_U2_rv | *Sch. pombe genomic DNA* |
| BB0537 | *ScMDH3ΔSKL*← | ScMdh3ΔSKL_U1_fw, ScMdh3ΔSKL_U1_rv | *S. cerevisiae genomic DNA* |
| BB0150 | *ScPYC2*→ | ScPYC2_U2_fw, ScPYC2_U2_rv | *S. cerevisiae genomic DNA* |

**Table S3. List of primers used in this study.** USER compatible ends are underlined.

| **Primer name** | **Sequence** | **Application** |
| --- | --- | --- |
| gRNAplfwd | ACCCAAUATCAGTTATTACCCTATGCG | amplification of a gRNA expression plasmid backbone without selection marker |
| gRNAplrev | ACGCGAUTGGTGCACTCTCAGTACAAT | amplification of a gRNA expression plasmid backbone without selection marker |
| ID399USERrev | ATTGGGUGCATAGGCCACTAGTGGATCTG | amplification of a loxP-flanked marker cassette |
| ID400USERfwd | ATCGCGUCAGCTGAAGCTTCGTACGC | amplification of a loxP-flanked marker cassette |
| GRE3gRNAfw | TACCAATCATAGATACGTACGTTTTAGAGCTAGAA | amplification of gRNA targeting *GRE3* gene |
| gRNArev | ^Pho^GATCATTTATCTTTCACTGCGGA | amplification of a gRNA vector |
| P1F (TJOS-62) | CGTGCGAUagggaacaaaagctggagct | amplification of a gRNA expression cassette for USER cloning into a multiple gRNA vector |
| P2F (TJOS-63) | AGTGCAGGUagggaacaaaagctggagct | amplification of a gRNA expression cassette for USER cloning into a multiple gRNA vector |
| P3F (TJOS-64) | ATCTGTCAUagggaacaaaagctggagct | amplification of a gRNA expression cassette for USER cloning into a multiple gRNA vector |
| P1R (TJOS-65) | CACGCGAUtaactaattacatgactcga | amplification of a gRNA expression cassette for USER cloning into a multiple gRNA vector |
| P2R (TJOS-66) | ACCTGCACUtaactaattacatgactcga | amplification of a gRNA expression cassette for USER cloning into a multiple gRNA vector |
| P3R (TJOS-67) | ATGACAGAUtaactaattacatgactcga | amplification of a gRNA expression cassette for USER cloning into a multiple gRNA vector |
| XYL3_1FW | AGTGCAGGUAAAACAATGACCACTACCCCATTTGA | amplification of the *P. stipitis* *XYL3* gene for cloning into integrative vector |
| XYL3_1REV | CGTGCGAUTCAGTGTTTCAATTCACTTTCCA | amplification of the *P. stipitis XYL3* gene for cloning into integrative vector |
| TAL1_1FW | AGTGCAGGUAAAACAATGTCCTCCAACTCCCTTGA | amplification of the *P. stipitis TAL1* gene for cloning into integrative vector |
| TAL1_1REV | CGTGCGAUTCAGAATCTGGCTTCCAATTGTT | amplification of the *P. stipitis TAL1* gene for cloning into integrative vector |
| tkl1_U2_fw | ATCTGTCAUAAAACAATGACTCAATTCACTGACATTG | amplification of *S. cerevisiae TKL1* gene for cloning into integrative vector |
| tkl1_U2_rv | CACGCGAUTCAGAAAGCTTTTTTCAAAGGAG | amplification of *S. cerevisiae TKL1* gene for cloning into integrative vector |
| RPE1_1FW | AGTGCAGGUAAAACAATGGTCAAACCAATTATAGC | amplification of *S. cerevisiae RPE1* gene for cloning into integrative vector |
| RPE1_1REV | CGTGCGAUTCAATCTAGCAAATCTCTAGAAC | amplification of *S. cerevisiae RPE1* gene for cloning into integrative vector |
| RKI1_2FW | ATCTGTCAUAAAACAATGGCTGCCGGTGTCCCAAA | amplification of *S. cerevisiae RKI1* gene for cloning into integrative vector |
| RKI1_2REV | CGTGCGAUTCACTTTTCGGTAACTTCAACAC | amplification of *S. cerevisiae RKI1* gene for cloning into integrative vector |
| PsSUT1_1FW | AGTGCAGGUAAAACAATGTCTTCTCAAGATATTCC | amplification of the *P. stipitis SUT1* gene for cloning into integrative vector |
| PsSUT1_1RE | CGTGCGAUTCAAACATGTTCGTCAACAGGCT | amplification of the *P. stipitis SUT1* gene for cloning into integrative vector |
| XIclos_2FW | ATCTGTCAUAAAACAATGAAGAACTACTTCCCAAA | amplification of synthetic *Clostridium phytofermentans* *XylA* gene for cloning into integrative vector |
| XIclos_2REV | CACGCGAUTCATCTGAACAAAATGTTGTTAA | amplification of synthetic *Clostridium phytofermentans XylA* gene for cloning into integrative vector |
| CpXylAgreFW | TGTAATATAAATCGTAAAGGAAAATTGGAAATTTTTAACGgcacacaccatagcttcaaa | amplification of P*_TEF1_*-*XylA* expression cassette for replacement of *GRE3* ORF |
| CpXylAgreREV | TTGTTCATATCGTCGTTGAGTATGGATTTTACTGGCTGGActtcgagcgtcccaaaac | amplification of P*_TEF1_*-*XylA* expression cassette for replacement of *GRE3* ORF |
| GREverFW | GACGCAGATACTGTAAATGC | verification of P*_TEF1_*-*XylA* insertion into *GRE3* locus |
| GREverREV | GCTTGATTCTACAACCACGT | verification of PTEF1-XylA insertion into GRE3 locus |
| ScPYC2_U2_fw | ATCTGTCAUAAAACAATGAGCAGTAGCAAGAAATTG | amplification of *PYC2* gene for cloning into integrative vector |
| ScPYC2_U2_rv | CACGCGAUTTACTTTTTTTGGGATGGG | amplification of *PYC2* gene for cloning into integrative vector |
| SpMae1_U2_fw | ATCTGTCAUAAAACAATGGGTGAACTCAAGGAAATC | amplification of *SpMae1* gene for cloning into integrative vector |
| SpMae1_U2_rv | CACGCGAUTTAAACGCTTTCATGTTCAC | amplification of *SpMae1* gene for cloning into integrative vector |
| ScMdh3deltaSKL_U1_fw | AGTGCAGGUAAAACAATGGTCAAAGTCGCAATTC | amplification of *MDH3* gene lacking peroxisomal targeting sequence gene for cloning into integrative vector |
| ScMdh3deltaSKL_U1_rv | CGTGCGAUTTAAGAGTCTAGGATGAAACTCTTG | amplification of *MDH3* gene lacking peroxisomal targeting sequence gene for cloning into integrative vector |
| PTEF1->_U2_rv | ATGACAGAUTTGTAATTAAAACTTAG | amplification of P*_TDH3_*-P*_TEF1_* biobrick for cloning into integrative vector |
| PTDH3_rv | ACCTGCACUTTTGTTTGTTTATGTGTGTTTATTC | amplification of P*_TDH3_*-P*_TEF1_* biobrick for cloning into integrative vector |
| PPGK1_fw | cgtgcgauggaagtaccttcaaaga | amplification of P*_PGK1_* biobrick for cloning into an integrative vector |
| PPGK1_rv | atgacagauttgttttatatttgttg | amplification of P*_PGK1_* biobrick for cloning into an integrative vector |
| ADH1_test_fw | GAAATTCGCTTATTTAGAAGTGTC | verification of a gene expression cassette cloning into EasyClone vectors |
| CYC1_test_rv | CTCCTTCCTTTTCGGTTAGAG | verification of a gene expression cassette cloning into EasyClone vectors |
| ID2220_vec_DW_out | CCTGCAGGACTAGTGCTGAG | verification of EasyClone vector chromosomal integration |
| ID2221_vec_UP_out | GTTGACACTTCTAAATAAGCGAATTTC | verification of EasyClone vector chromosomal integration |
| ID903 X-3-up-out-sq | TGACGAATCGTTAGGCACAG | verification of site X-3 chromosomal integration |
| ID904 X-3-down-out-sq | CCGTGCAATACCAAAATCG | verification of site X-3 chromosomal integration |
| ID905 X-4-up-out-sq | CTCACAAAGGGACGAATCCT | verification of site X-4 chromosomal integration |
| ID906 X-4-down-out-sq | GACGGTACGTTGACCAGAG | verification of site X-4 chromosomal integration |
| ID907 XI-1-up-out-sq | CTTAATGGGTAGTGCTTGACACG | verification of site XI-1 chromosomal integration |
| ID908 XI-1-down-out-sq | GAAGACCCATGGTTCCAAGGA | verification of site XI-1 chromosomal integration |
| ID911 XI-3-up-out-sq | GTGCTTGATTTGCGTCATTC | verification of site XI-5 chromosomal integration |
| ID912 XI-3-down-out-sq | CACATTGAGCGAATGAAACG | verification of site XI-5 chromosomal integration |
| ID893 XII-2-up-out-sq | CGAAGAAGGCCTGCAATTC | verification of site XII-2 chromosomal integration |
| ID894 XII-2-down-out-sq | GGCCCTGATAAGGTTGTTG | verification of site XII-2 chromosomal integration |
| ID899 XII-5-up-out-sq | CCACCGAAGTTGATTTGCTT | verification of site XII-5 chromosomal integration |
| ID900 XII-5-down-out-sq | GTGGGAGTAAGGGATCCTGT | verification of site XII-5 chromosomal integration |

**Table S4. Genes with aminoacid mutations found across all strains**.

| **S288c genes** | | | | **Plasmid genes** | **Ethanol Red-specific genes*** |
| --- | --- | --- | --- | --- | --- |
| YAL039C | YFR007W | YHL038C | YJR091C | *Ps*SUT1 | NW130CH00330 |
| YAL063C | YFR032C | YHL039W | YJR092W | *Cp*XylA | NW130GN00100 |
| YAR050W | YFR041C | YHL040C | YJR126C |  | NW130GN00110 |
| YBL005W-B | YGL003C | YHL047C | YJR140C |  | NW130GN00130 |
| YBL016W | YGL005C | YHR005C | YJR151C |  | NW130HT00100 |
| YBL100W-B | YGL006W-A | YHR007C | YKR056W |  | NW130ID00120 |
| YBL102W | YGL009C | YHR009C | YKR059W |  | NW130IO00100 |
| YBR011C | YGL013C | YHR012W | YKR105C |  | NW130JH00100 |
| YBR085C-A | YGL014W | YHR014W | YLR086W |  |  |
| YBR140C | YGL015C | YHR101C | YLR146C |  |  |
| YCL019W | YGL016W | YHR172W | YLR151C |  |  |
| YCL024W | YGL017W | YIL019W | YLR256W |  |  |
| YCL027W | YGL020C | YIL082W-A | YLR300W |  |  |
| YCL030C | YGL027C | YIL115C | YLR341W |  |  |
| YCL032W | YGL028C | YIL177C | YLR362W |  |  |
| YCL033C | YGL032C | YIR028W | YLR389C |  |  |
| YCL037C | YGL033W | YJL004C | YLR429W |  |  |
| YCL038C | YGL035C | YJL005W | YML066C |  |  |
| YCL039W | YGL036W | YJL019W | YML109W |  |  |
| YCL042W | YGL039W | YJL020C | YMR013C |  |  |
| YCL045C | YGL045W | YJL025W | YMR045C |  |  |
| YCL048W | YGL047W | YJL027C | YMR050C |  |  |
| YCL049C | YGL066W | YJL028W | YMR052W |  |  |
| YCL050C | YGL071W | YJL029C | YMR065W |  |  |
| YCL051W | YGL093W | YJL033W | YMR119W |  |  |
| YCL069W | YGL111W | YJL035C | YMR139W |  |  |
| YCL073C | YGL116W | YJL037W | YMR155W |  |  |
| YDL003W | YGL141W | YJL039C | YMR196W |  |  |
| YDL005C | YGL148W | YJL041W | YMR209C |  |  |
| YDL013W | YGL158W | YJL042W | YMR218C |  |  |
| YDL019C | YGL173C | YJL049W | YMR234W |  |  |
| YDL022C-A | YGL240W | YJL050W | YMR308C |  |  |
| YDL024C | YGL254W | YJL051W | YNL018C |  |  |
| YDL025C | YGR001C | YJL054W | YNL023C |  |  |
| YDL027C | YGR002C | YJL055W | YNL063W |  |  |
| YDL029W | YGR004W | YJL057C | YNL083W |  |  |
| YDL030W | YGR008C | YJL058C | YNL088W |  |  |
| YDL031W | YGR010W | YJL061W | YNL270C |  |  |
| YDL033C | YGR014W | YJL062W | YNL294C |  |  |
| YDL035C | YGR021W | YJL069C | YNL311C |  |  |
| YDL054C | YGR023W | YJL070C | YNL336W |  |  |

| YDL058W | YGR024C | YJL073W | YNR006W |  |  |
| --- | --- | --- | --- | --- | --- |
| YDL097C | YGR029W | YJL076W | YNR013C |  |  |
| YDL124W | YGR030C | YJL077C | YNR051C |  |  |
| YDR001C | YGR036C | YJL082W | YOL036W |  |  |
| YDR006C | YGR038W | YJL083W | YOL075C |  |  |
| YDR012W | YGR040W | YJL085W | YOL078W |  |  |
| YDR022C | YGR041W | YJL087C | YOL156W |  |  |
| YDR023W | YGR042W | YJL088W | YOR057W |  |  |
| YDR026C | YGR043C | YJL089W | YOR120W |  |  |
| YDR027C | YGR049W | YJL090C | YOR123C |  |  |
| YDR028C | YGR055W | YJL091C | YOR124C |  |  |
| YDR030C | YGR061C | YJL092W | YOR126C |  |  |
| YDR033W | YGR062C | YJL093C | YOR127W |  |  |
| YDR034C | YGR067C | YJL094C | YOR128C |  |  |
| YDR034C-D | YGR068C | YJL095W | YOR137C |  |  |
| YDR041W | YGR070W | YJL097W | YOR188W |  |  |
| YDR042C | YGR071C | YJL098W | YOR192C-B |  |  |
| YDR043C | YGR077C | YJL208C | YOR343W-B |  |  |
| YDR049W | YGR081C | YJL222W | YOR348C |  |  |
| YDR051C | YGR087C | YJL225C | YOR355W |  |  |
| YDR055W | YGR088W | YJR001W | YOR357C |  |  |
| YDR056C | YGR089W | YJR002W | YOR368W |  |  |
| YDR057W | YGR090W | YJR010C-A | YOR383C |  |  |
| YDR059C | YGR094W | YJR012C | YPL002C |  |  |
| YDR060W | YGR095C | YJR015W | YPL003W |  |  |
| YDR066C | YGR096W | YJR016C | YPL006W |  |  |
| YDR073W | YGR097W | YJR019C | YPL058C |  |  |
| YDR074W | YGR098C | YJR024C | YPL131W |  |  |
| YDR077W | YGR099W | YJR030C | YPL158C |  |  |
| YDR080W | YGR100W | YJR031C | YPL180W |  |  |
| YDR085C | YGR109W-B | YJR032W | YPL215W |  |  |
| YDR093W | YGR112W | YJR033C | YPL227C |  |  |
| YDR097C | YGR113W | YJR035W | YPL257W |  |  |
| YDR103W | YGR117C | YJR036C | YPR001W |  |  |
| YDR114C | YGR119C | YJR039W | YPR002W |  |  |
| YDR143C | YGR120C | YJR040W | YPR003C |  |  |
| YDR150W | YGR121C | YJR041C | YPR005C |  |  |
| YDR170W-A | YGR134W | YJR045C | YPR006C |  |  |
| YDR200C | YGR143W | YJR053W | YPR007C |  |  |
| YDR210C-D | YGR161W-B | YJR054W | YPR008W |  |  |
| YDR223W | YGR177C | YJR055W | YPR010C |  |  |
| YDR277C | YGR197C | YJR058C | YPR013C |  |  |
| YDR334W | YGR295C | YJR059W | YPR018W |  |  |

| YDR379W | YHL006C | YJR060W | YPR021C |  |  |
| --- | --- | --- | --- | --- | --- |
| YDR393W | YHL008C | YJR061W | YPR022C |  |  |
| YDR419W | YHL014C | YJR062C | YPR024W |  |  |
| YDR456W | YHL017W | YJR078W | YPR027C |  |  |
| YEL069C | YHL025W | YJR080C | YPR055W |  |  |
| YER096W | YHL026C | YJR083C | YPR074C |  |  |
| YFL002W-A | YHL027W | YJR084W | YPR095C |  |  |
| YFL022C | YHL028W | YJR085C | YPR158W-B |  |  |
| YFL038C | YHL030W | YJR088C | YPR198W |  |  |
| YFR005C | YHL032C | YJR089W | YPR199C |  |  |
| YFR006W | YHL035C | YJR090C | YPR200C |  |  |

*Gene names are according to the annotated Ethanol Red genome assembly used as reference [6].

**Table S12. Average* copy numbers for the engineered xylose pathway genes in each strain**.

| **Gene** | **E.R.** | **V1** | **EV1** | **EV2** | **EV4** | **EV5** | **EV6** | **EV7** | **EV8** | **EV9** | **EV10** | **EV11** | **EV12** |
| --- | --- | --- | --- | --- | --- | --- | --- | --- | --- | --- | --- | --- | --- |
| *PsSUT1* | 0,0 | 1,9 | 0,0 | 1,4 | 0,0 | 0,0 | 0,0 | 1,6 | 1,6 | 0,0 | 1,7 | 1,7 | 1,7 |
|  | ±0,0 | ±0,1 | ±0,0 | ±0,2 | ±0,0 | ±0,0 | ±0,0 | ±0,2 | ±0,2 | ±0,0 | ±0,3 | ±0,2 | ±0,3 |
|  |  |  |  |  |  |  |  |  |  |  |  |  |  |
| *PsXYL3* | 0,0 | 2,8 | 4,2 | 1,3 | 3,1 | 4,0 | 3,2 | 2,0 | 1,8 | 3,4 | 2,4 | 1,9 | 2,4 |
|  | ±0,0 | ±0,3 | ±0,4 | ±0,2 | ±0,3 | ±0,4 | ±0,4 | ±0,3 | ±0,2 | ±0,4 | ±0,3 | ±0,3 | ±0,3 |
|  |  |  |  |  |  |  |  |  |  |  |  |  |  |
| *PsTAL1* | 0,0 | 2,6 | 2,4 | 2,9 | 2,4 | 2,1 | 3,3 | 2,5 | 2,7 | 2,8 | 2,7 | 2,6 | 3,2 |
|  | ±0,0 | ±0,2 | ±0,2 | ±0,2 | ±0,2 | ±0,2 | ±0,3 | ±0,3 | ±0,3 | ±0,3 | ±0,3 | ±0,3 | ±0,4 |
|  |  |  |  |  |  |  |  |  |  |  |  |  |  |
| *RKI1* | 1,9 | 4,7 | 4,2 | 4,6 | 4,3 | 4,8 | 4,5 | 4,0 | 3,9 | 4,0 | 4,2 | 4,3 | 5,5 |
|  | ±0,1 | ±0,3 | ±0,3 | ±0,2 | ±0,3 | ±0,4 | ±0,3 | ±0,2 | ±0,2 | ±0,4 | ±0,3 | ±0,4 | ±0,3 |
|  |  |  |  |  |  |  |  |  |  |  |  |  |  |
| *RPE1* | 1,7 | 3,6 | 3,6 | 2,5 | 2,8 | 3,8 | 3,0 | 2,8 | 3,0 | 3,7 | 3,2 | 2,7 | 3,1 |
|  | ±0,1 | ±0,2 | ±0,3 | ±0,3 | ±0,3 | ±0,4 | ±0,3 | ±0,3 | ±0,3 | ±0,3 | ±0,2 | ±0,2 | ±0,3 |
|  |  |  |  |  |  |  |  |  |  |  |  |  |  |
| *TKL1* | 2,0 | 3,6 | 4,0 | 4,2 | 4,8 | 4,2 | 4,1 | 4,5 | 4,5 | 4,5 | 5,2 | 4,2 | 4,8 |
|  | ±0,5 | ±0,3 | ±0,3 | ±0,3 | ±0,4 | ±0,3 | ±0,3 | ±0,4 | ±0,2 | ±0,5 | ±0,4 | ±0,4 | ±0,3 |
|  |  |  |  |  |  |  |  |  |  |  |  |  |  |
| *Cp*XylA | 0,0 | 6,5 | 6,6 | 4,1 | 4,7 | 6,0 | 5,3 | 4,9 | 5,2 | 5,4 | 5,6 | 5,3 | 6,2 |
|  | ±0,0 | ±0,4 | ±0,7 | ±0,5 | ±0,6 | ±0,6 | ±0,7 | ±0,4 | ±0,5 | ±0,5 | ±0,8 | ±0,5 | ±0,5 |
|  |  |  |  |  |  |  |  |  |  |  |  |  |  |

*Average of the values per base position ±SD over the gene length.

**Supplementary references**

1. Jessop‐Fabre MM, Jakočiūnas T, Stovicek V, Dai Z, Jensen MK, Keasling JD, et al. EasyClone‐MarkerFree: A vector toolkit for marker‐less integration of genes into *Saccharomyces cerevisiae* via CRISPR‐Cas9. Biotechnol J. 2016;11:1110–7.

2. Stovicek V, Borodina I, Forster J. CRISPR–Cas system enables fast and simple genome editing of industrial *Saccharomyces cerevisiae* strains. Metab Eng Commun. 2015;2:13–22.

3. Ronda C, Maury J, Jakočiu̅nas T, Baallal Jacobsen SA, Germann SM, Harrison SJ, et al. CrEdit: CRISPR mediated multi-loci gene integration in *Saccharomyces cerevisiae*. Microb Cell Factories. 2015;14:97.

4. Stovicek V, Borja GM, Forster J, Borodina I. EasyClone 2.0: expanded toolkit of integrative vectors for stable gene expression in industrial *Saccharomyces cerevisiae* strains. J Ind Microbiol Biotechnol. 2015;42:1519–31.

5. Partow S, Siewers V, Bjørn S, Nielsen J, Maury J. Characterization of different promoters for designing a new expression vector in Saccharomyces cerevisiae. Yeast Chichester Engl. 2010;27:955–64.

6. Kang K, Bergdahl B, Machado D, Dato L, Han T-L, Li J, et al. Linking genetic, metabolic, and phenotypic diversity among Saccharomyces cerevisiae strains using multi-omics associations. GigaScience. 2019;8.
