# Supplementary material for: Rational and evolutionary engineering of Saccharomyces cerevisiae for production of dicarboxylic acids from lignocellulosic biomass and exploring genetic mechanisms of the yeast tolerance to the biomass hydrolysate
Source: Biotechnol Biofuels Bioprod. 2022 Feb 27;15:22. doi: 10.1186/s13068-022-02121-1 (PMC8882276; doi:10.1186/s13068-022-02121-1)
Supplement: Supplementary file 2 — Additional file 2: Fig. S1. Adaptive evolution of the xylose consuming strain XylC2 V1 in the SSL, parallel lines. Time course of each parallel evolution line. Color bars indicate increased concentration of the SSL. a MM SSL evolution lines. b YE SSL evolution lines. Asterisk indicates the EV3 evolution line that was terminated for poor tolerance in the 70% SSL. Fig. S2. Evolution of the xylose consuming strain, single isolates. Growth profiles of the evolved strains cultivated in YPX medium. Fig. S3. Evolution of the xylose consuming strain. Growth of the selected SSL-tolerant strains XylC2 EV6_4 and EV9_5 in the a YE SSL and b MM SSL at pH = 4.5. The experiment was performed in duplicates, error bars represent standard deviation (N = 2). Fig. S4. Growth of the tolerant XylC2 EV6_4 and its dicarboxylic acid producing derivative XylC2 EV6_4 MA in 70% and 60% YE SSL. The experiments were performed in two parallel shake flasks, error bars represent standard deviation (N = 2). Fig. S5. Production of dicarboxylic acids by fermentation of the SSL in bioreactors, second parallel bioreactor experiment. Consumption of the carbon sources present in the SSL and biomass formation by the engineered XylC2 6_4 MA strain (on the left) and production of dicarboxylic acids from the SSL at pH = 4.5 (on the right). Fig. S6. Mapping of the mutations to the respective loci on the reference genome of S288c. Panel A: overview of the distribution across the whole genome (Chr I to XVI, top) of the total mutations (blue, bottom) and the aminoacid-changing mutations (red, middle) found across all strains. Panels B–R: Distribution of the variants, mapped to each individual chromosome, in each of the strains (V1 parent and EV1–EV12, indicated on the left). Mutations are highlighted in orange if heterozygous or in red if homozygous, and referred to the S288c genomic sequence (annotation is shown on top, genes are represented by green boxes. Only few gene names are visible due to limited space). [file 13068_2022_2121_MOESM2_ESM.pptx]

## Slide 1
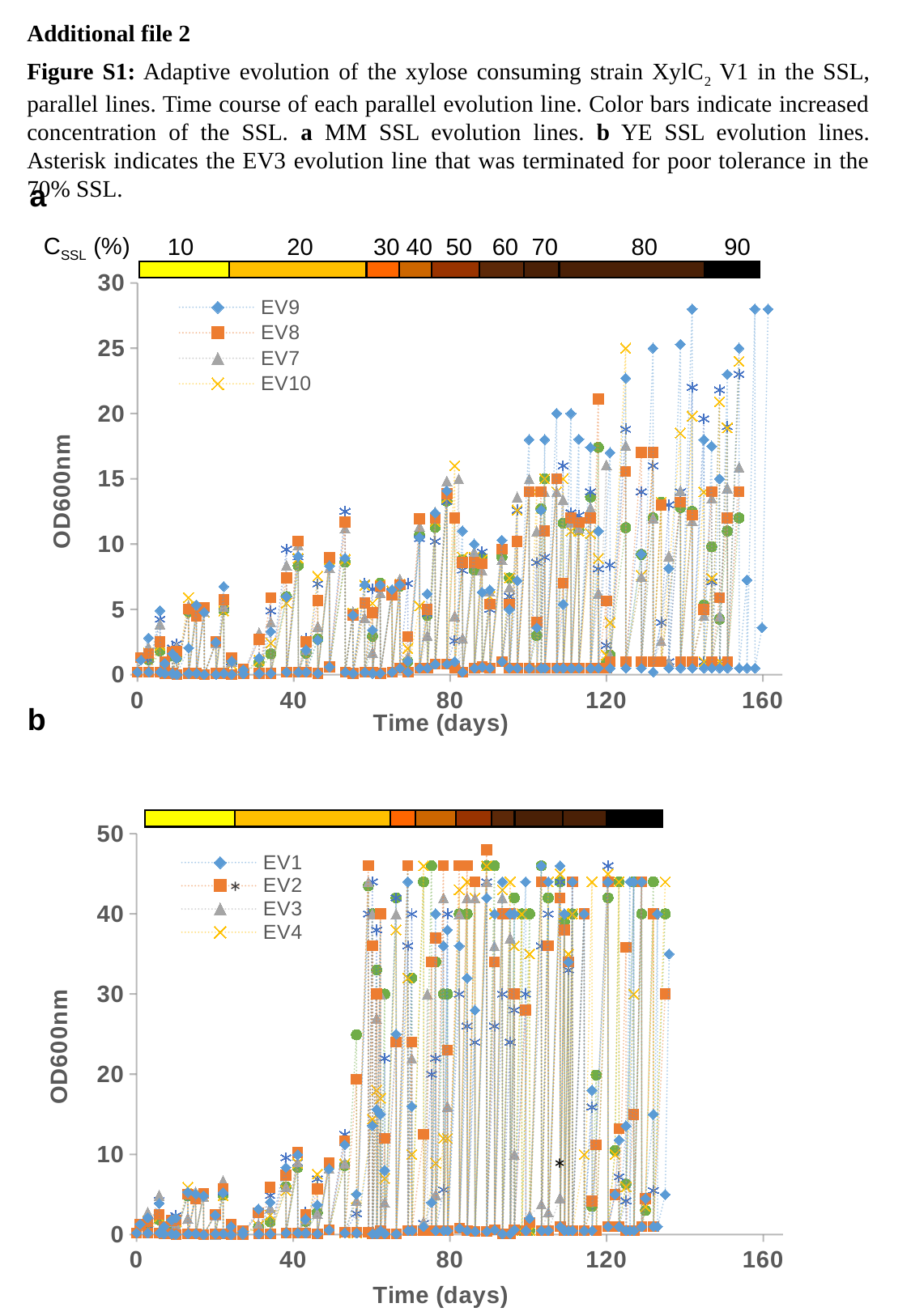

Additional file 2
Figure S1: Adaptive evolution of the xylose consuming strain XylC2 V1 in the SSL, parallel lines. Time course of each parallel evolution line. Color bars indicate increased concentration of the SSL. a MM SSL evolution lines. b YE SSL evolution lines. Asterisk indicates the EV3 evolution line that was terminated for poor tolerance in the 70% SSL.
a
 10 20 30 40 50 60 70 80 90
CSSL (%)
### Chart
| Category | | | | | | |
|---|---|---|---|---|---|---|
b
### Chart
| Category | | | | | | |
|---|---|---|---|---|---|---|*
*

## Slide 2
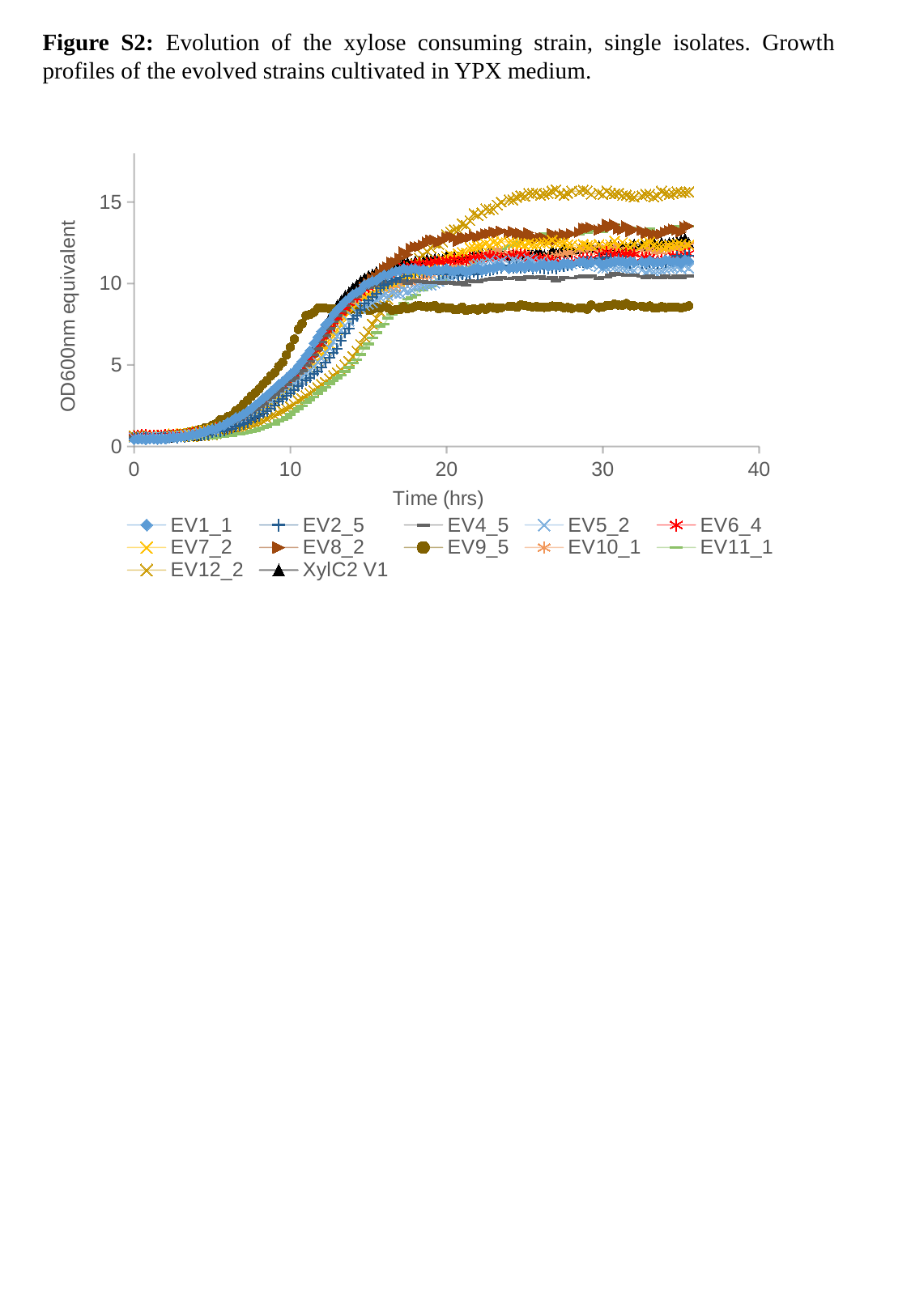

Figure S2: Evolution of the xylose consuming strain, single isolates. Growth profiles of the evolved strains cultivated in YPX medium.
### Chart
| Category | | | | | | | | | | | | |
|---|---|---|---|---|---|---|---|---|---|---|---|---|

## Slide 3
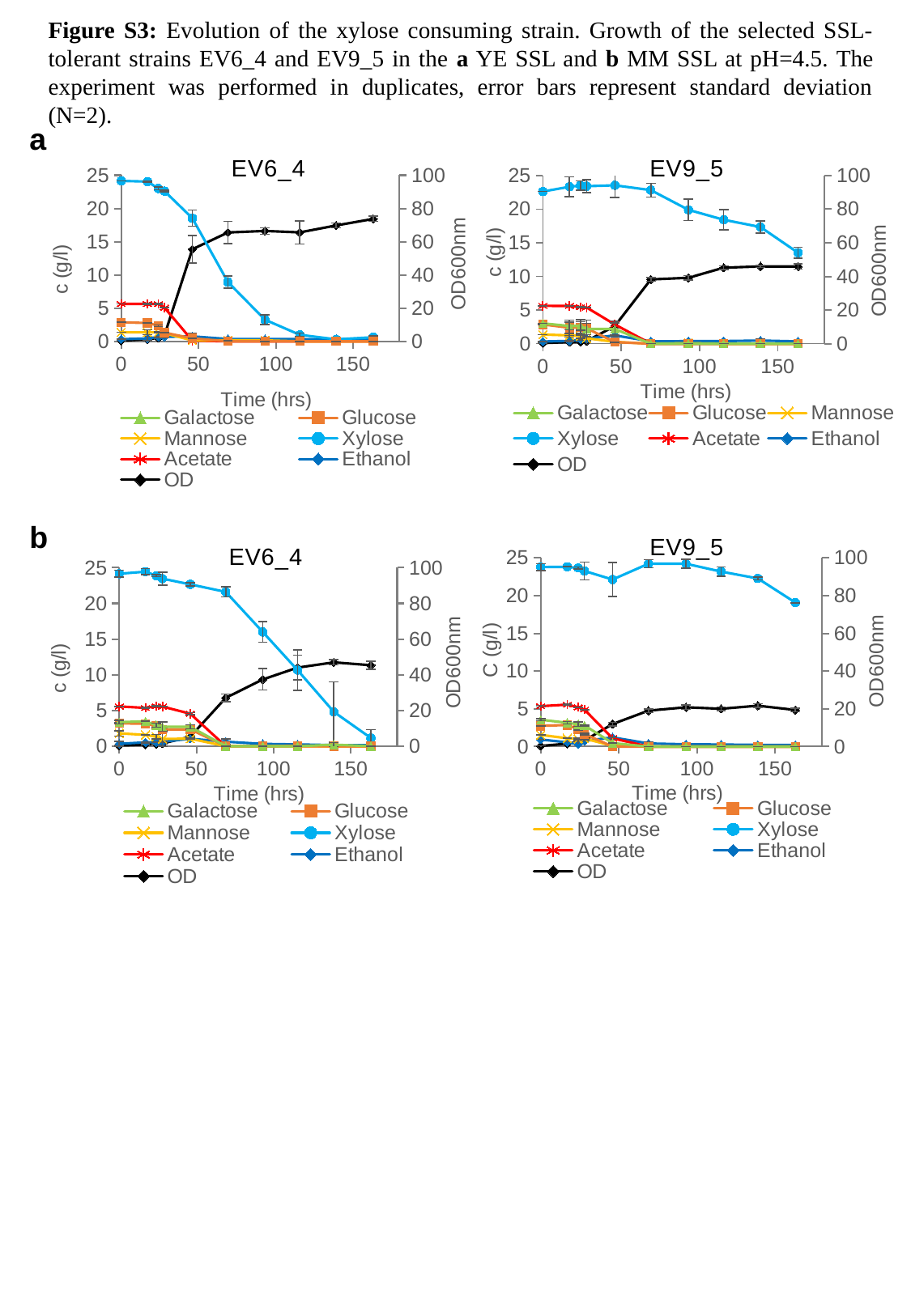

Figure S3: Evolution of the xylose consuming strain. Growth of the selected SSL-tolerant strains EV6_4 and EV9_5 in the a YE SSL and b MM SSL at pH=4.5. The experiment was performed in duplicates, error bars represent standard deviation (N=2).
### Chart: EV6_4
| Category | Galactose | Glucose | Mannose | Xylose | Acetate | Ethanol | |
|---|---|---|---|---|---|---|---|a
### Chart: EV9_5
| Category | Galactose | Glucose | Mannose | Xylose | Acetate | Ethanol | |
|---|---|---|---|---|---|---|---|
### Chart: EV6_4
| Category | Galactose | Glucose | Mannose | Xylose | Acetate | Ethanol | |
|---|---|---|---|---|---|---|---|b
### Chart: EV9_5
| Category | Galactose | Glucose | Mannose | Xylose | Acetate | Ethanol | |
|---|---|---|---|---|---|---|---|

## Slide 4
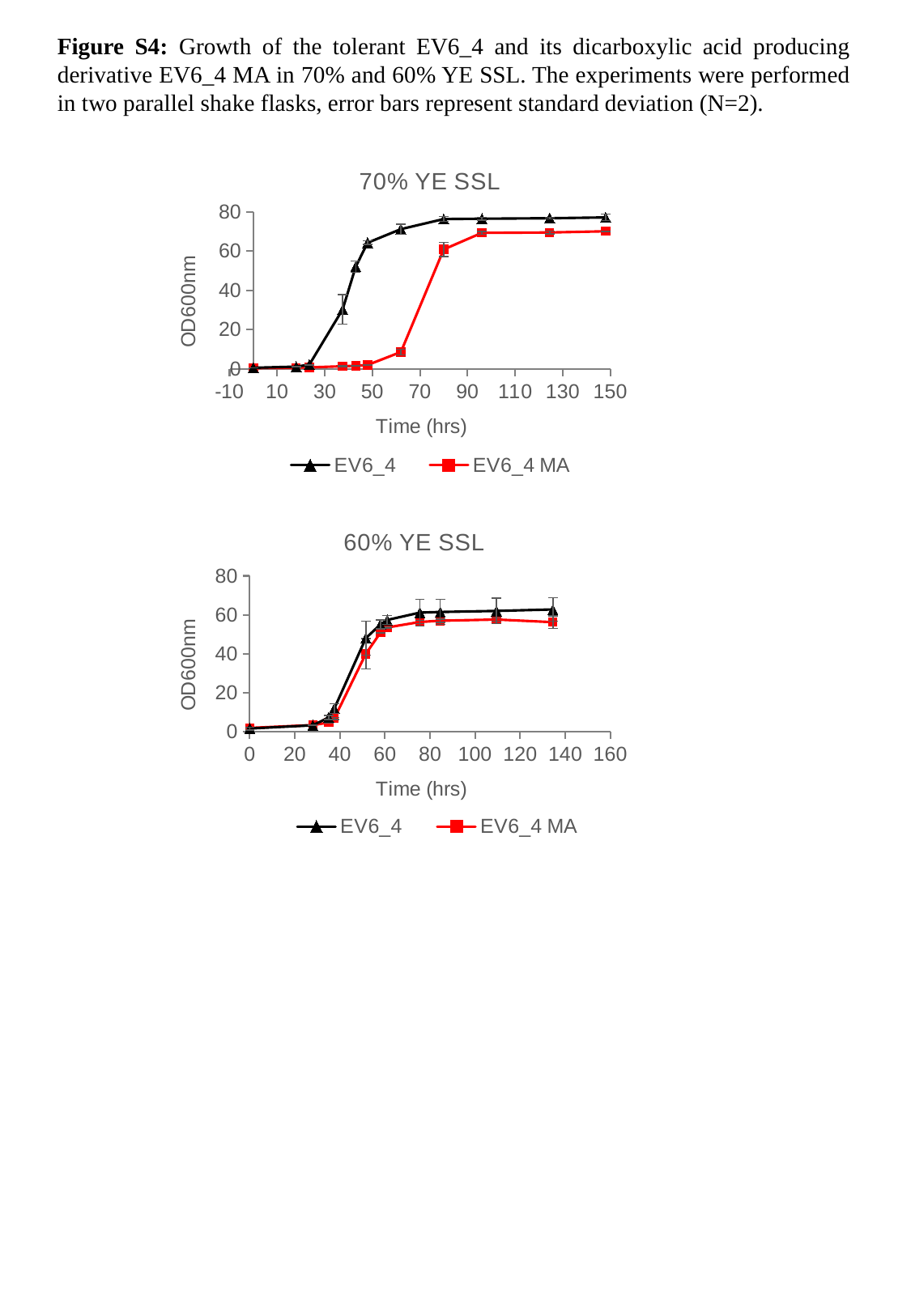

Figure S4: Growth of the tolerant EV6_4 and its dicarboxylic acid producing derivative EV6_4 MA in 70% and 60% YE SSL. The experiments were performed in two parallel shake flasks, error bars represent standard deviation (N=2).
### Chart: 70% YE SSL
| Category | | |
|---|---|---|
### Chart: 60% YE SSL
| Category | | |
|---|---|---|

## Slide 5
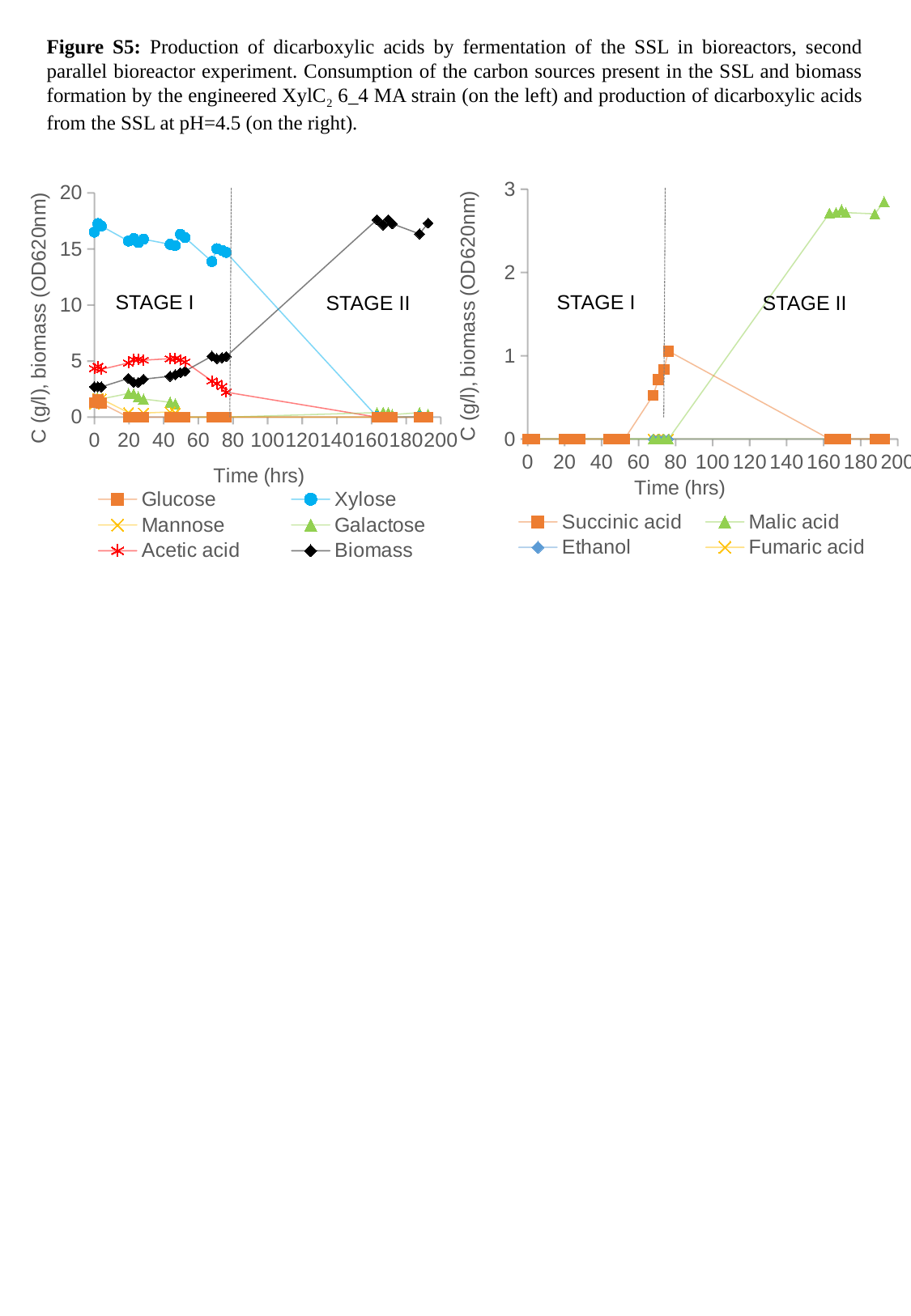

Figure S5: Production of dicarboxylic acids by fermentation of the SSL in bioreactors, second parallel bioreactor experiment. Consumption of the carbon sources present in the SSL and biomass formation by the engineered XylC2 6_4 MA strain (on the left) and production of dicarboxylic acids from the SSL at pH=4.5 (on the right).
### Chart
| Category | Glucose | Xylose | Mannose | Galactose | Acetic acid | Biomass |
|---|---|---|---|---|---|---|
### Chart
| Category | Succinic acid | Malic acid | Ethanol | Fumaric acid |
|---|---|---|---|---|STAGE I
STAGE I
STAGE II
STAGE II

## Slide 6
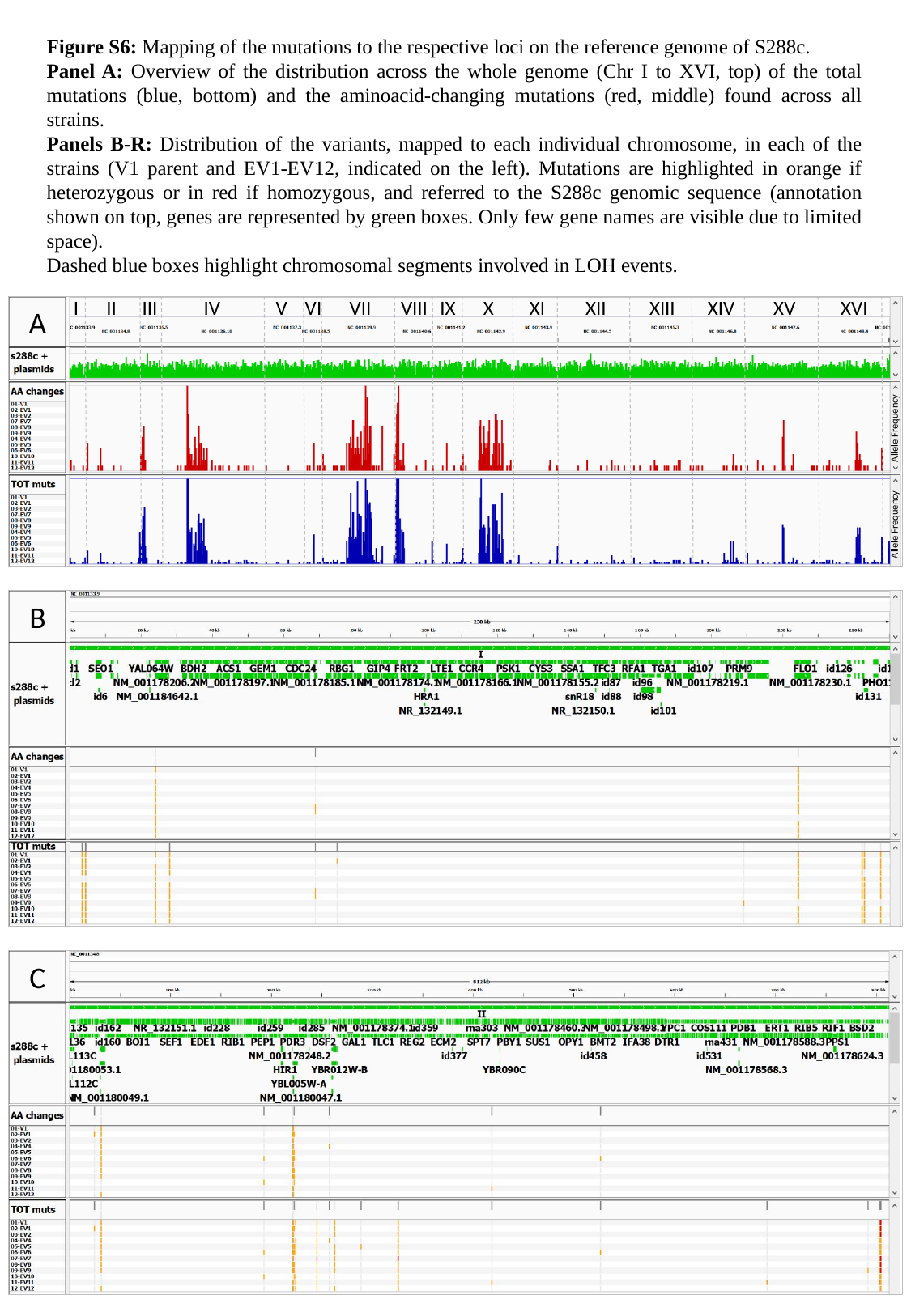

Figure S6: Mapping of the mutations to the respective loci on the reference genome of S288c.
Panel A: Overview of the distribution across the whole genome (Chr I to XVI, top) of the total mutations (blue, bottom) and the aminoacid-changing mutations (red, middle) found across all strains.
Panels B-R: Distribution of the variants, mapped to each individual chromosome, in each of the strains (V1 parent and EV1-EV12, indicated on the left). Mutations are highlighted in orange if heterozygous or in red if homozygous, and referred to the S288c genomic sequence (annotation shown on top, genes are represented by green boxes. Only few gene names are visible due to limited space).
Dashed blue boxes highlight chromosomal segments involved in LOH events.
I II III IV V VI VII VIII IX X XI XII XIII XIV XV XVI
A
Allele Frequency
Allele Frequency
B
C

## Slide 7
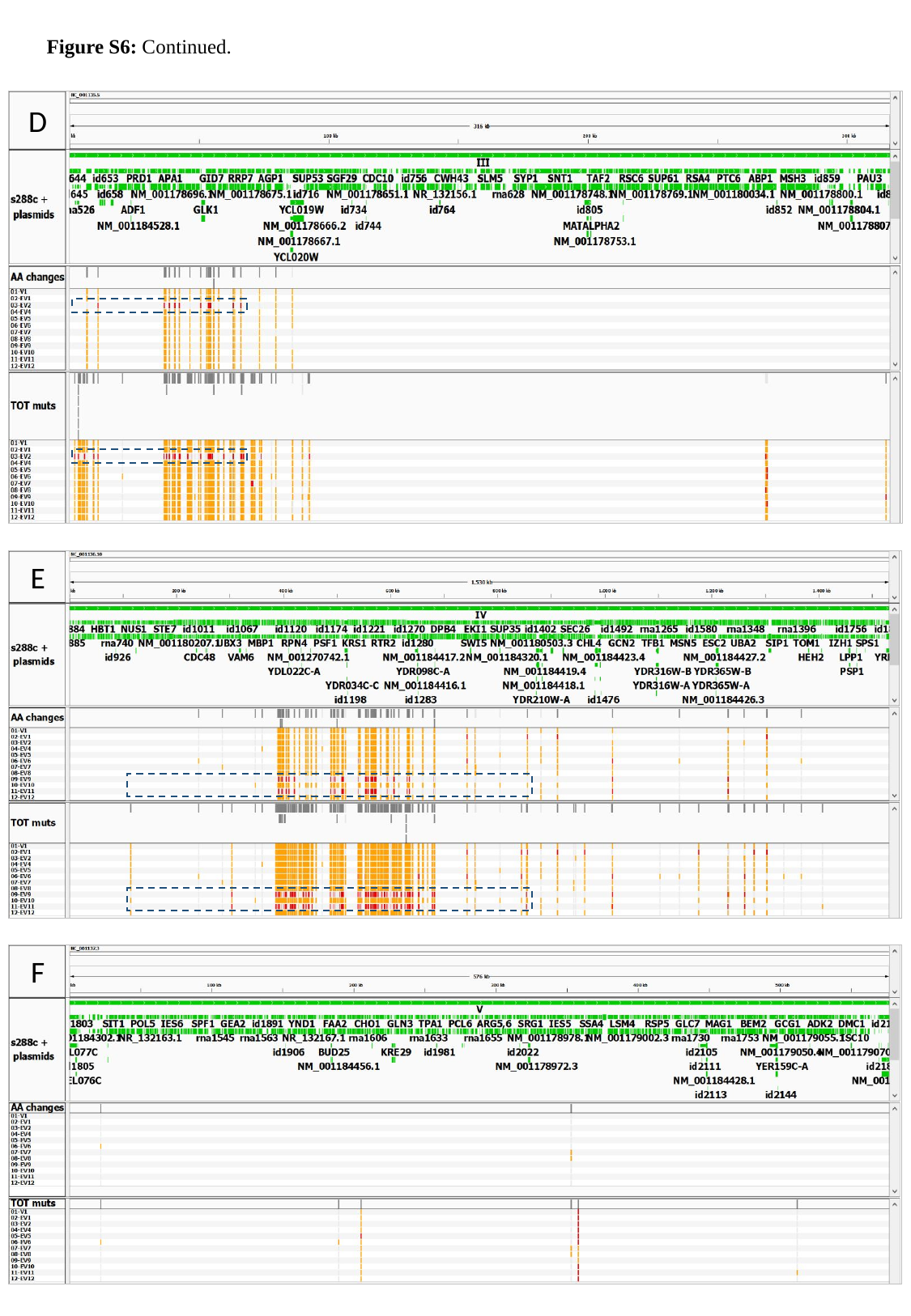

Figure S6: Continued.
D
E
F

## Slide 8
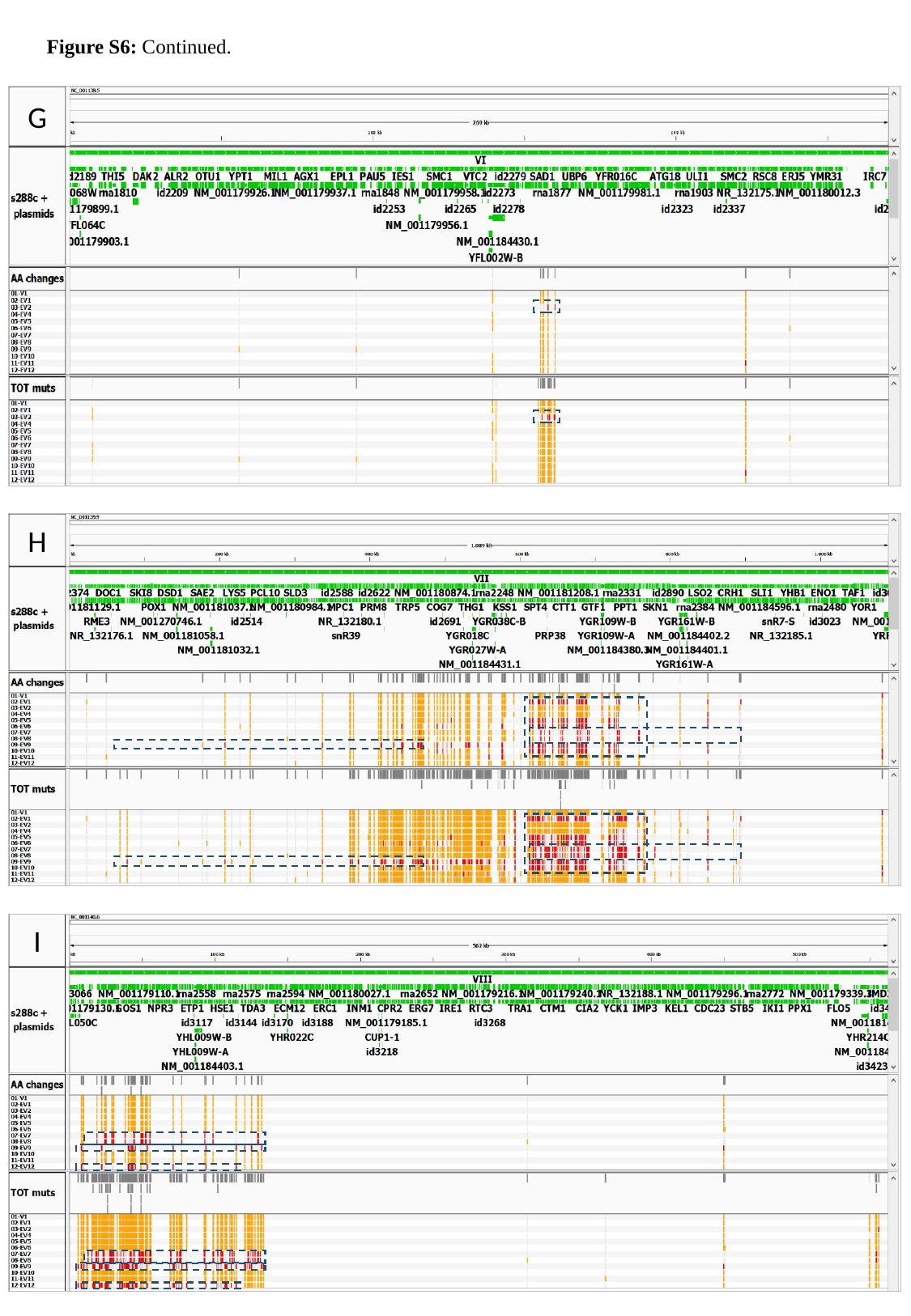

Figure S6: Continued.
G
H
I

## Slide 9
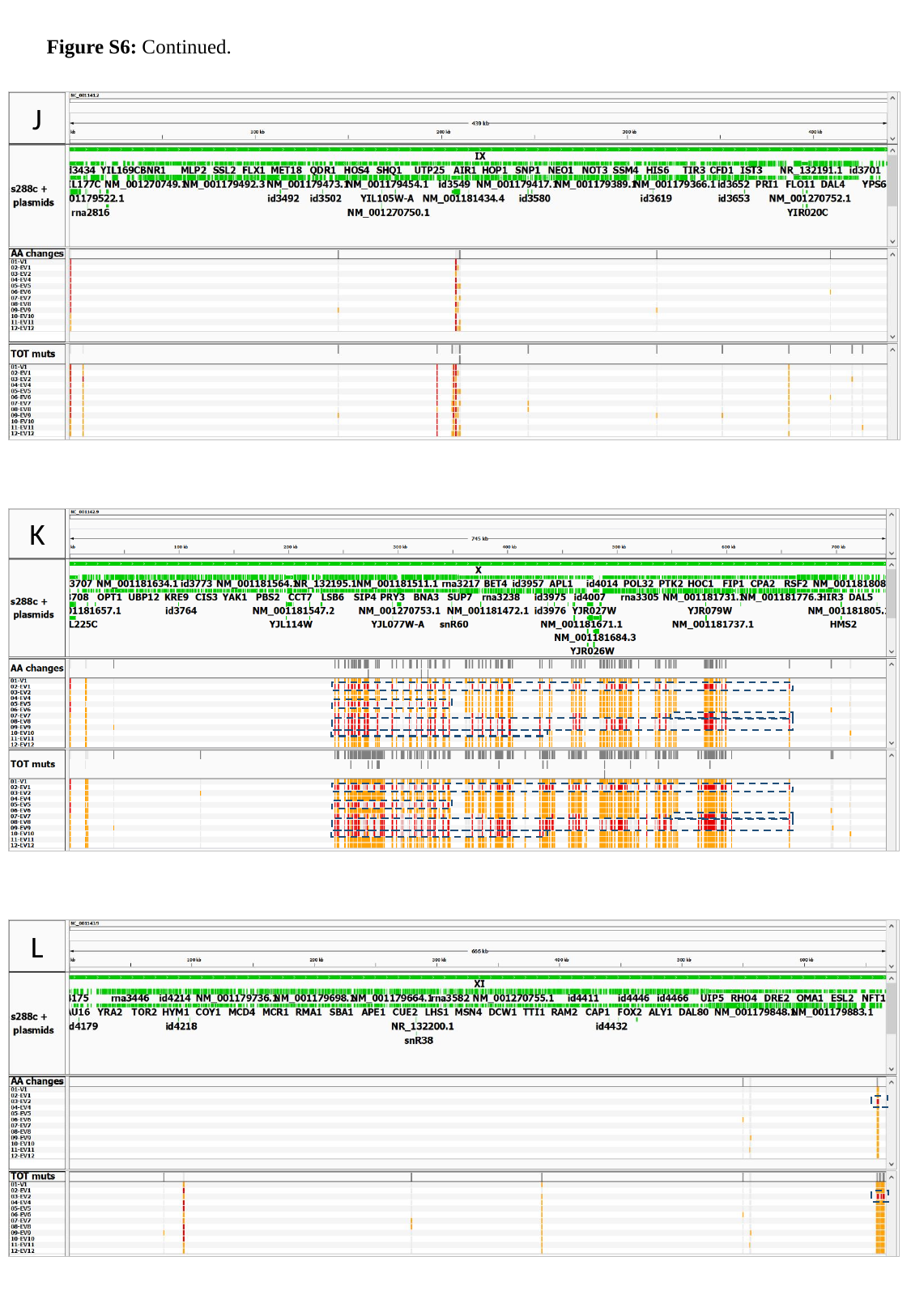

Figure S6: Continued.
J
K
L

## Slide 10
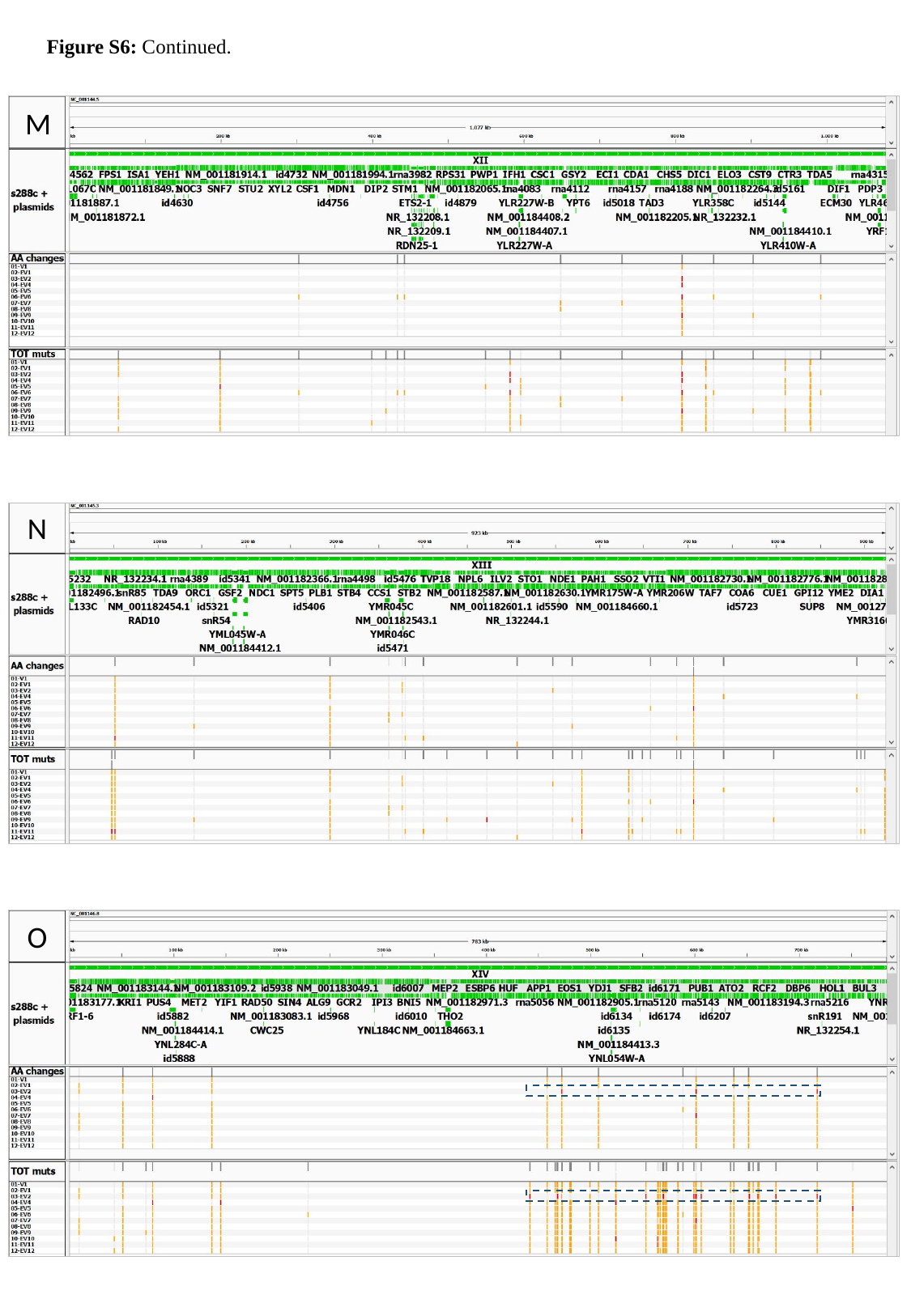

Figure S6: Continued.
M
N
O

## Slide 11
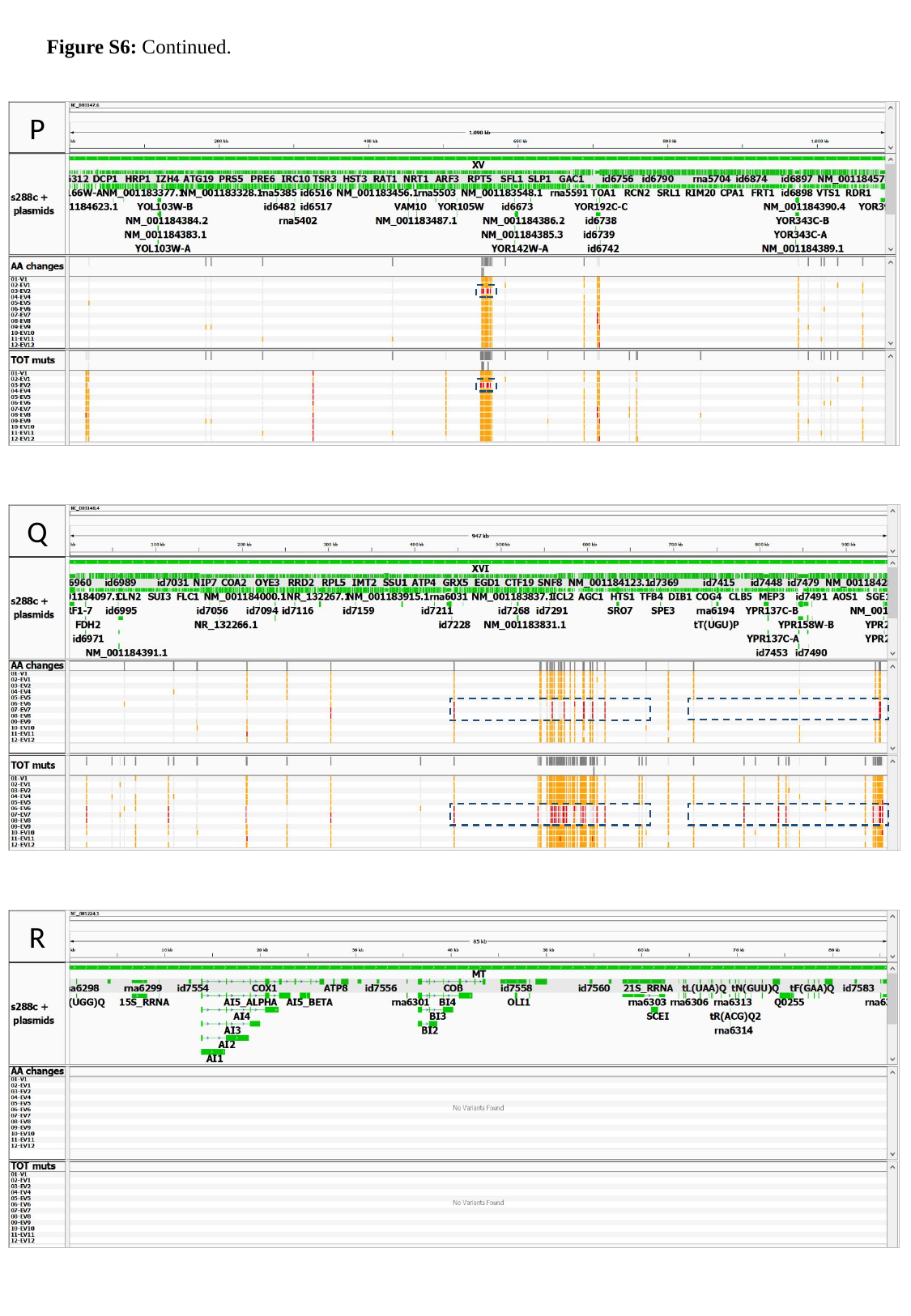

Figure S6: Continued.
P
Q
R

## Slide 12
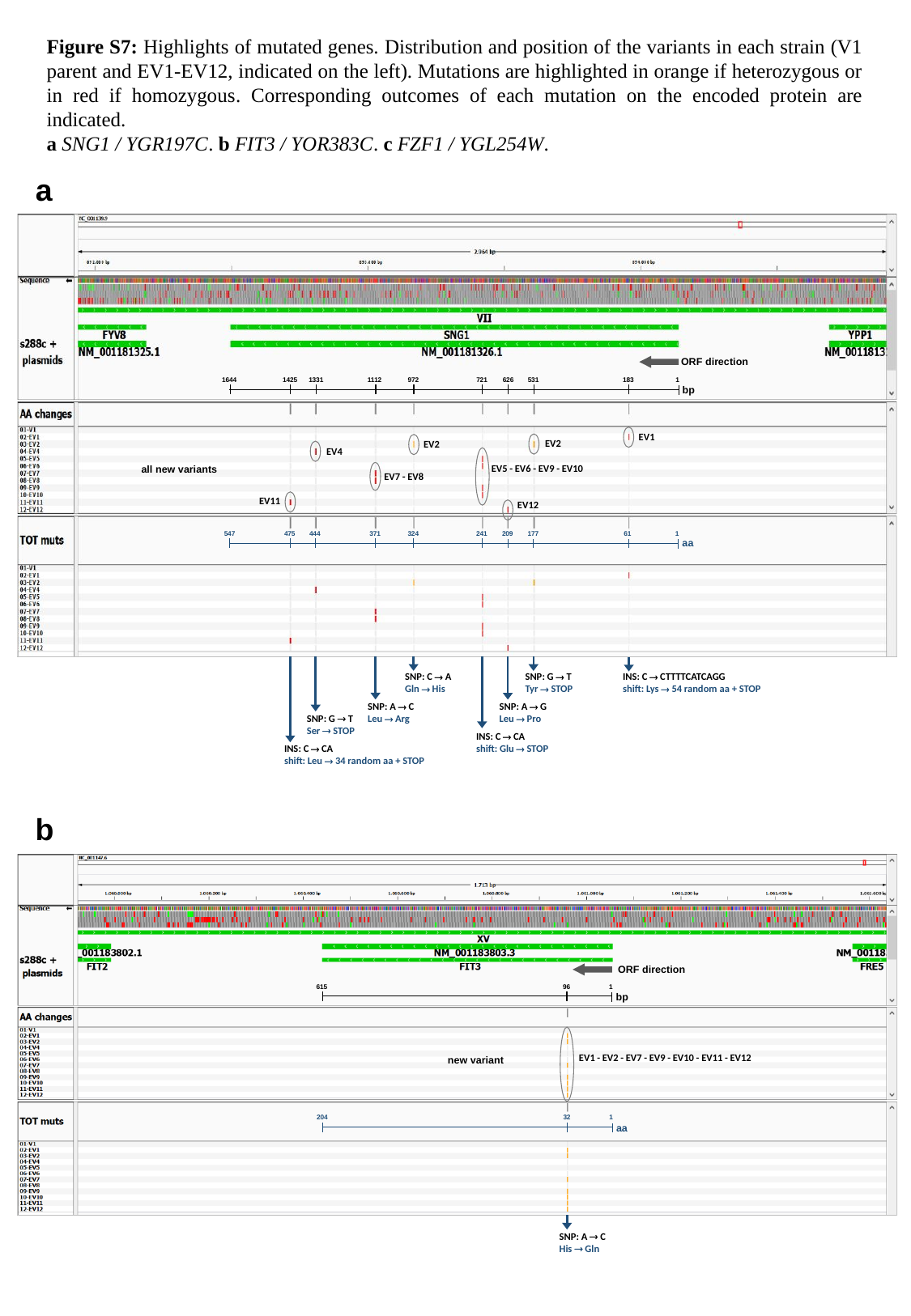

Figure S7: Highlights of mutated genes. Distribution and position of the variants in each strain (V1 parent and EV1-EV12, indicated on the left). Mutations are highlighted in orange if heterozygous or in red if homozygous. Corresponding outcomes of each mutation on the encoded protein are indicated.
a SNG1 / YGR197C. b FIT3 / YOR383C. c FZF1 / YGL254W.
a
ORF direction
1644
1425
1331
1112
972
721
626
531
183
1
bp
EV1
EV2
EV2
EV4
EV5 - EV6 - EV9 - EV10
EV7 - EV8
EV11
EV12
547
475
444
371
324
241
209
177
61
1
aa
SNP: C  A
Gln  His
SNP: G  T
Tyr  STOP
INS: C  CTTTTCATCAGG
shift: Lys  54 random aa + STOP
SNP: A  C
Leu  Arg
SNP: A  G
Leu  Pro
SNP: G  T
Ser  STOP
INS: C  CA
shift: Glu  STOP
INS: C  CA
shift: Leu  34 random aa + STOP
all new variants
b
ORF direction
615
96
1
bp
EV1 - EV2 - EV7 - EV9 - EV10 - EV11 - EV12
204
32
1
aa
SNP: A  C
His  Gln
new variant

## Slide 13
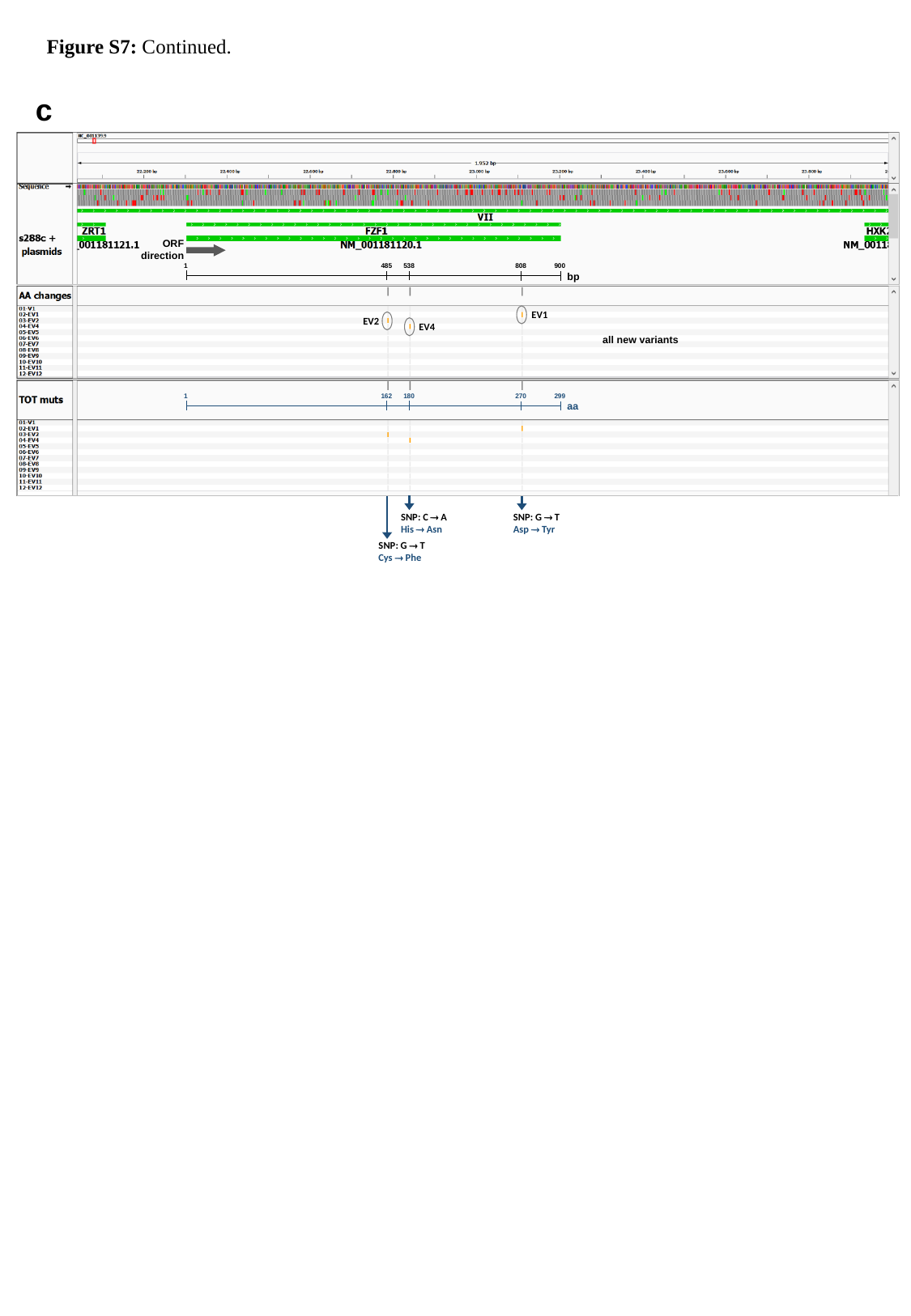

Figure S7: Continued.
c
ORF direction
1
485
538
808
900
bp
EV1
EV2
EV4
all new variants
1
162
180
270
299
aa
SNP: C  A
His  Asn
SNP: G  T
Asp  Tyr
SNP: G  T
Cys  Phe

## Slide 14
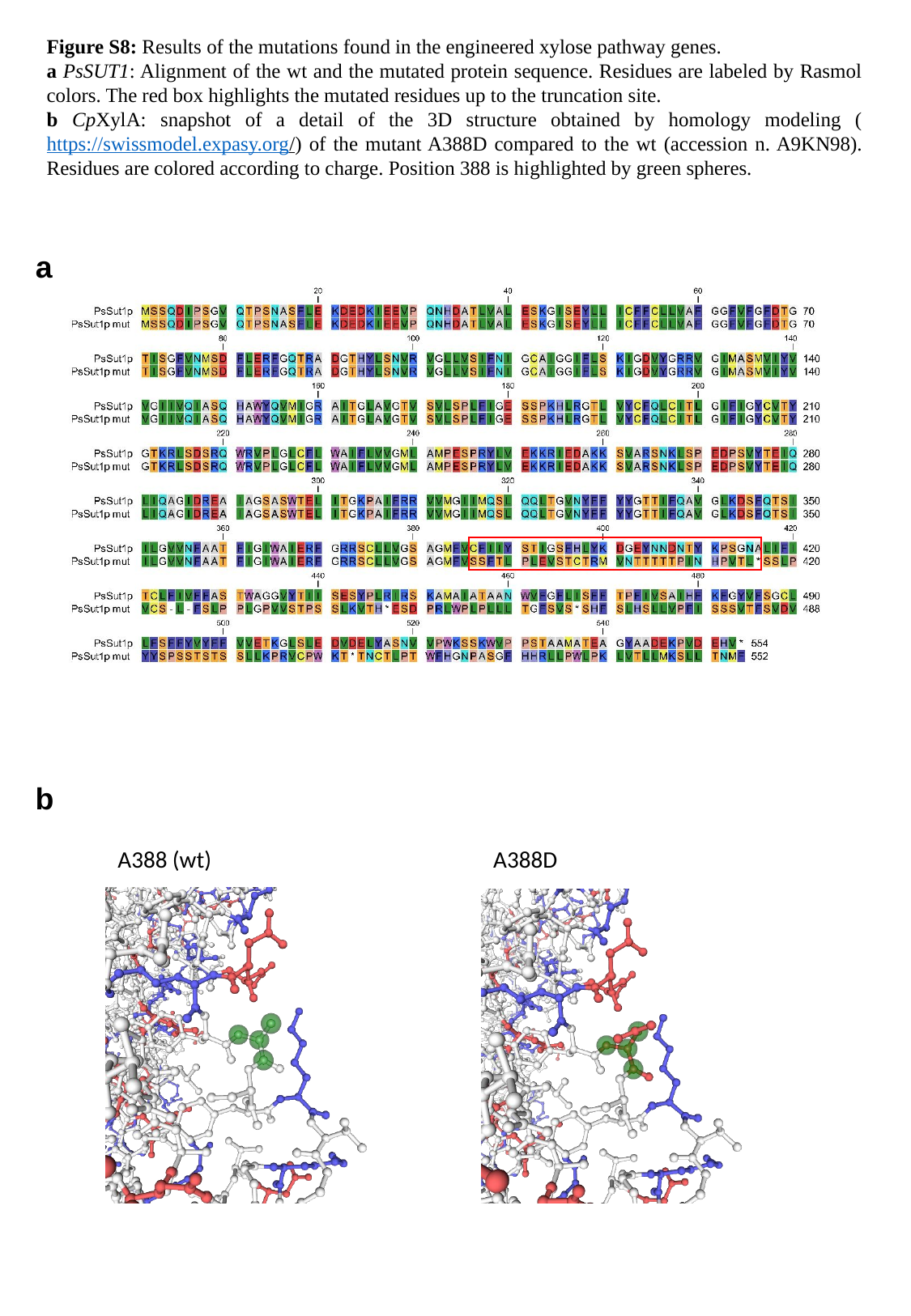

Figure S8: Results of the mutations found in the engineered xylose pathway genes.
a PsSUT1: Alignment of the wt and the mutated protein sequence. Residues are labeled by Rasmol colors. The red box highlights the mutated residues up to the truncation site.
b CpXylA: snapshot of a detail of the 3D structure obtained by homology modeling (https://swissmodel.expasy.org/) of the mutant A388D compared to the wt (accession n. A9KN98). Residues are colored according to charge. Position 388 is highlighted by green spheres.
a
b
A388 (wt)
A388D
